# Supplementary figures and images for: Computational Analysis of MDR1 Variants Predicts Effect on Cancer Cells via their Effect on mRNA Folding
Source: PLoS Comput Biol. 2024 Dec 26;20(12):e1012685. doi: 10.1371/journal.pcbi.1012685 (PMC11670953; doi:10.1371/journal.pcbi.1012685)

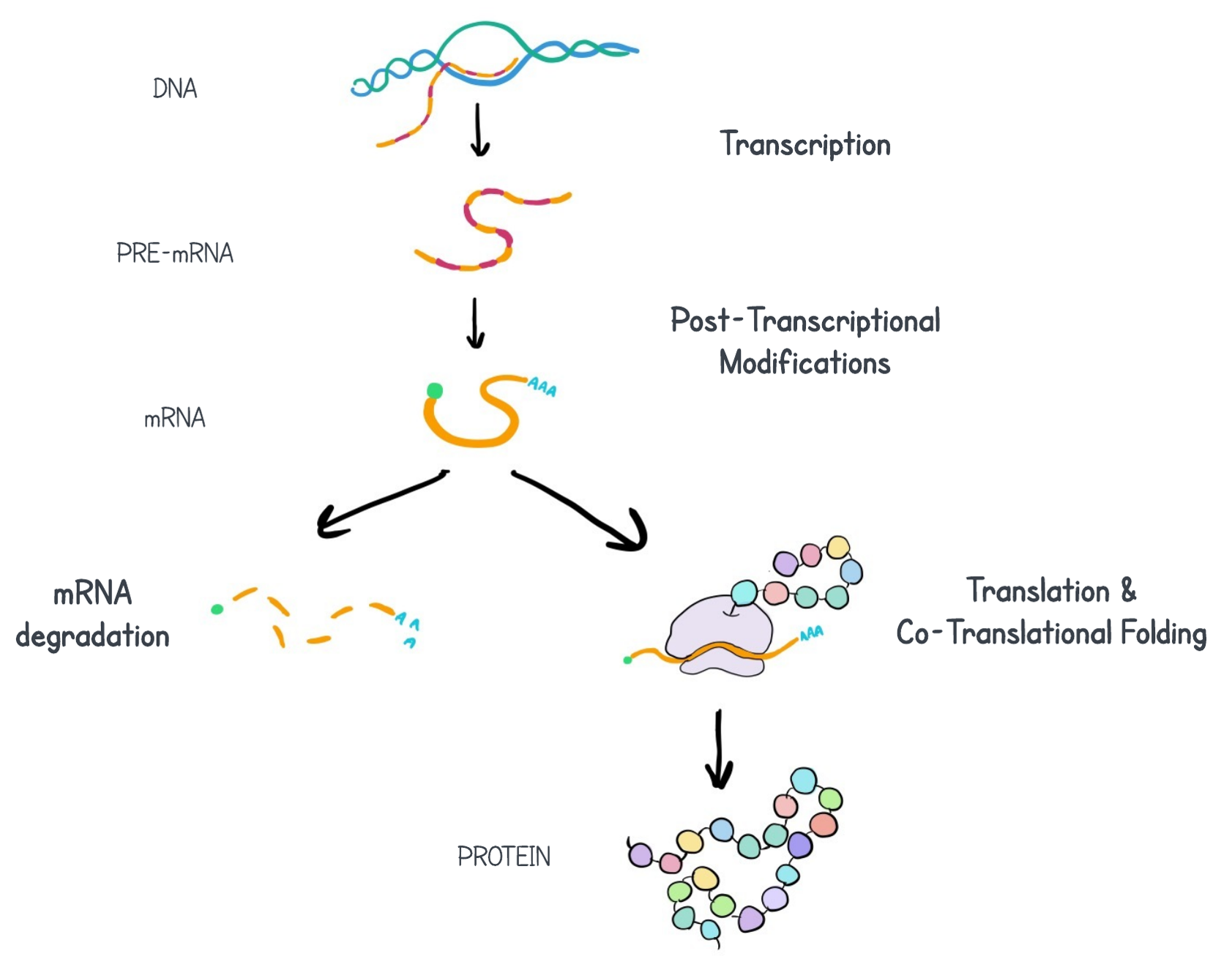

Supplement: S1 Fig — Transcription: A region of the double helix of the DNA is unwound and a pre-mRNA sequence is transcribed using one of the DNA strands as templet. Splicing: The pre-mRNA is edited and introns are removed from the it. Additionally, a 5’ cap and 3’ poly-A tail are added to the mRNA. Translation: The ribosome synthesizes a protein according to the mRNA templet. During the translation process the nascent protein initiates the formation of secondary and tertiary structures. mRNA degradation: mRNAs undergo degradation and are broken to smaller fragments, mainly by ribonucleases. (PNG) [file pcbi.1012685.s001.png]

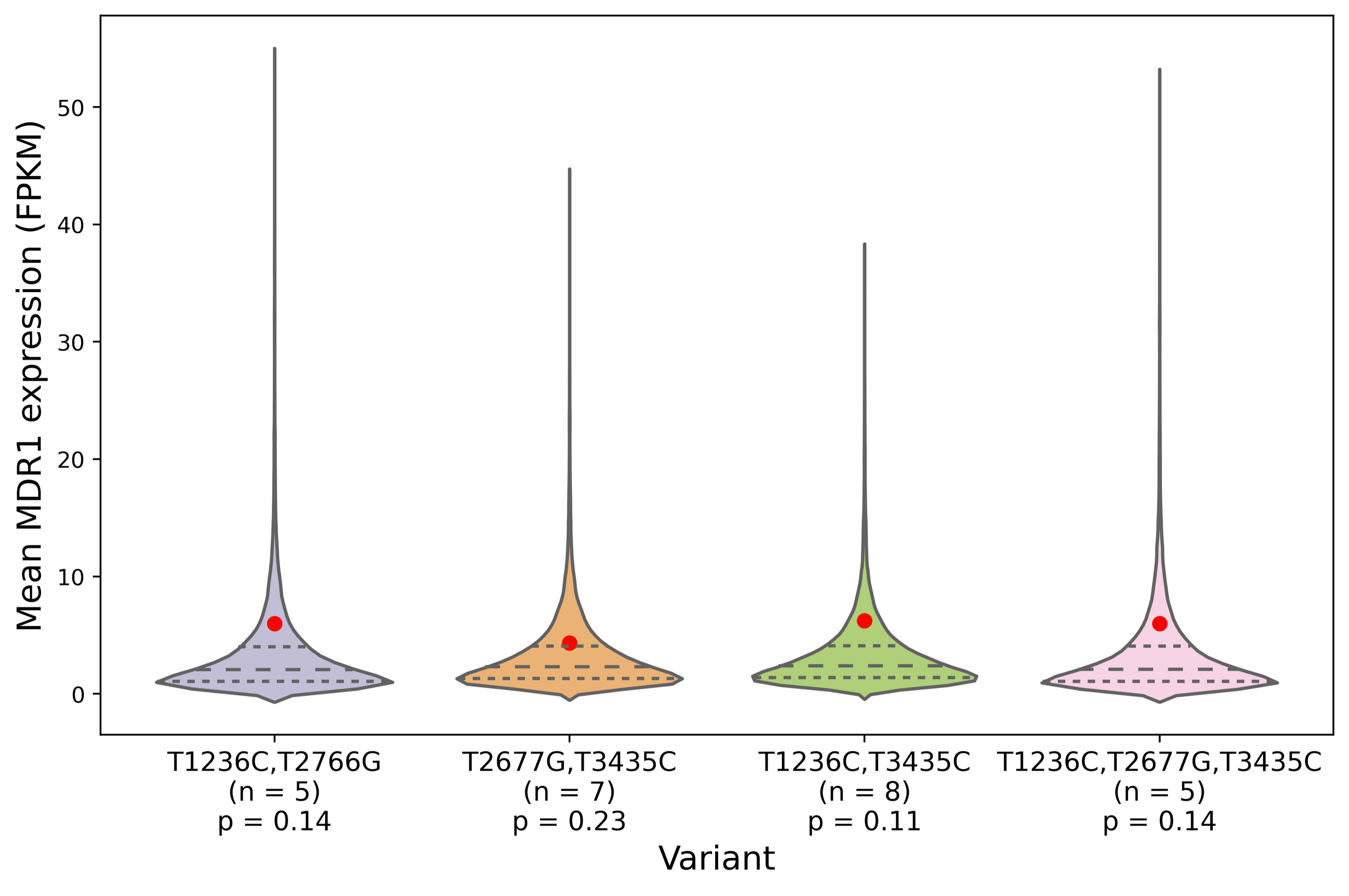

Supplement: S2 Fig — a) T1236C & T2677G; b) T1236C & T3435C; c) T2677G & T3435C;. d) T1236C, T2677G & T3435C. Red dot represents the mean MDR1 expression level of carriers, while violin plots depict the distribution of mean MDR1 expression levels among 100,000 randomly chosen non-carriers. Both groups are matched in size for accurate comparison. (PNG) [file pcbi.1012685.s002.png]

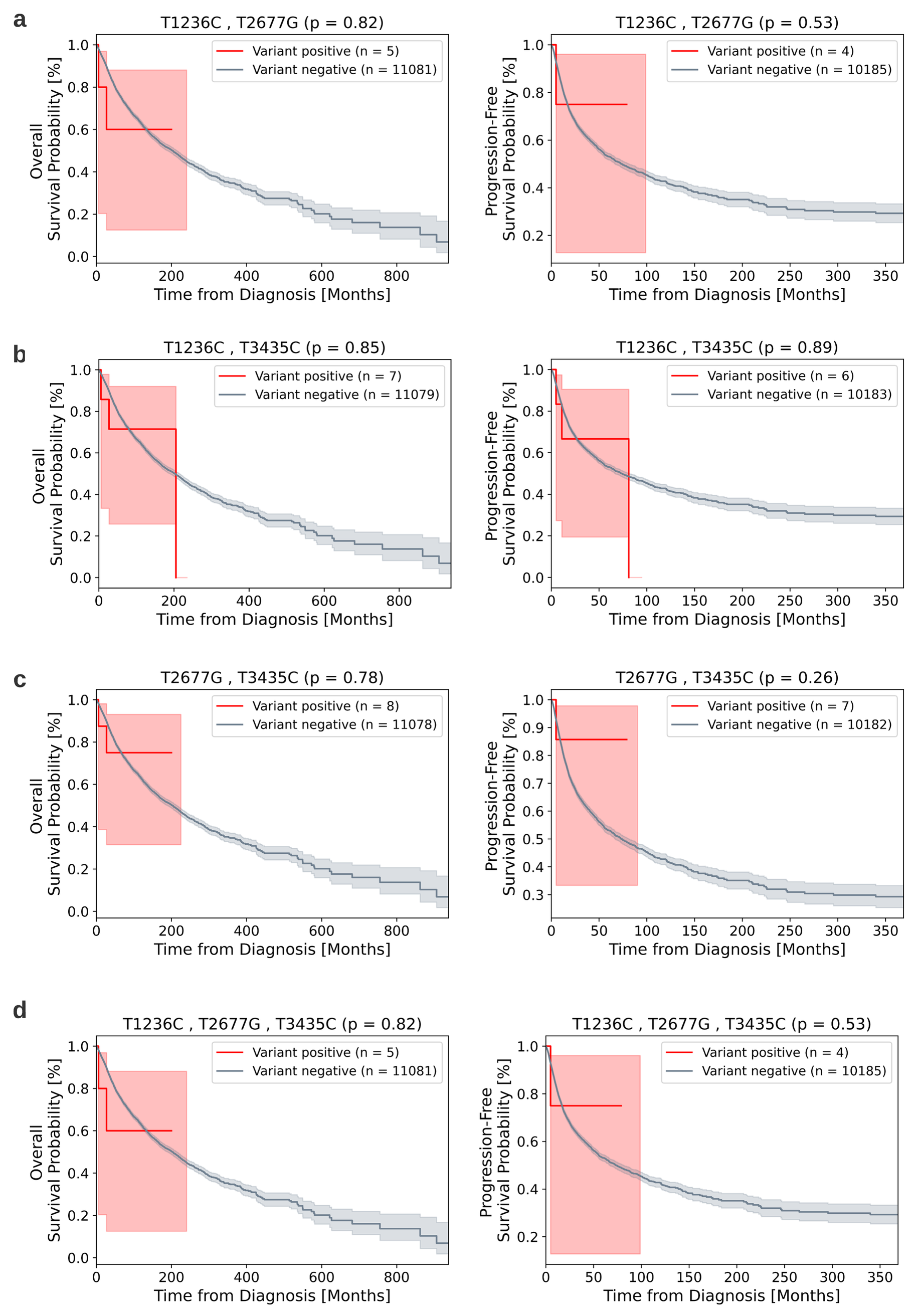

Supplement: S3 Fig — a) T1236C & T2677G; b) T1236C & T3435C; c) T2677G & T3435C;. d) T1236C, T2677G & T3435C. Left column: overall survival. Right column: progression-free survival. (PNG) [file pcbi.1012685.s003.png]

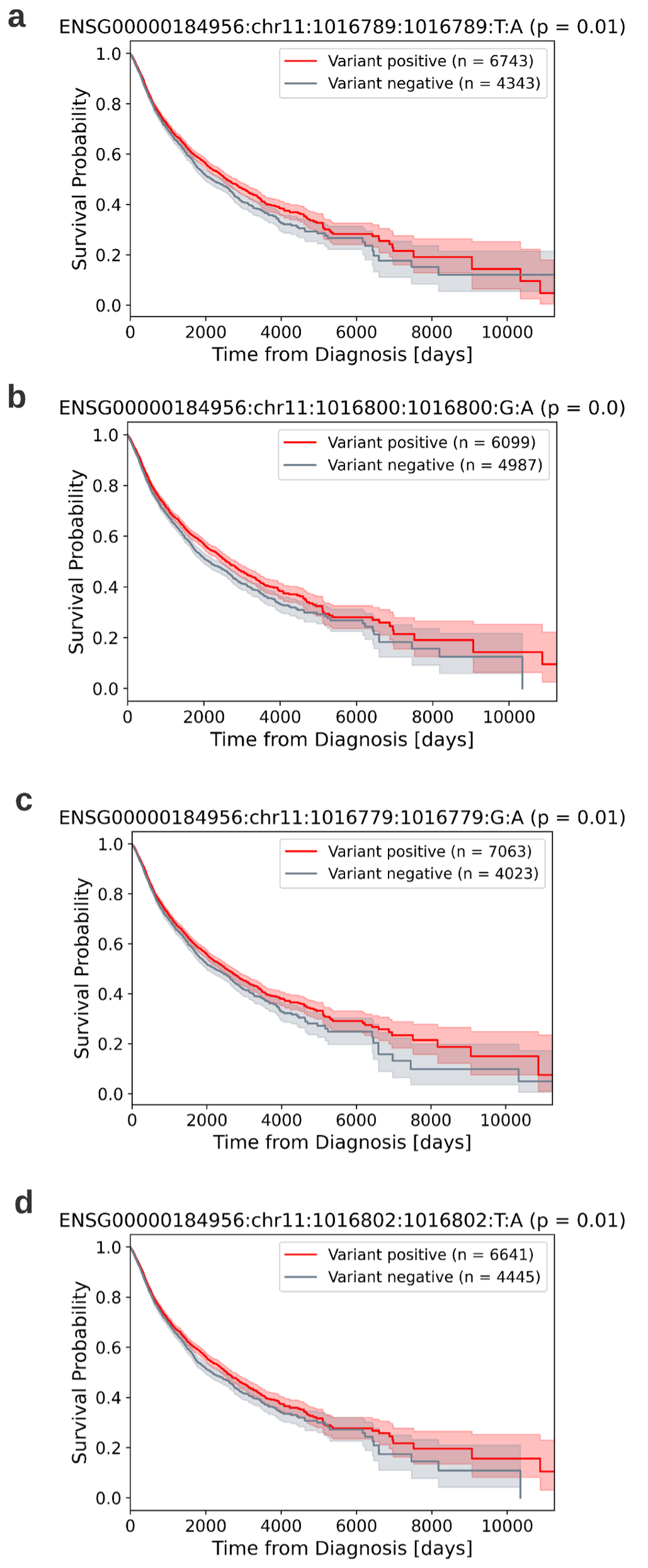

Supplement: S4 Fig — These variants are detected in 75% or more of T1236C positive patients. (PNG) [file pcbi.1012685.s004.png]

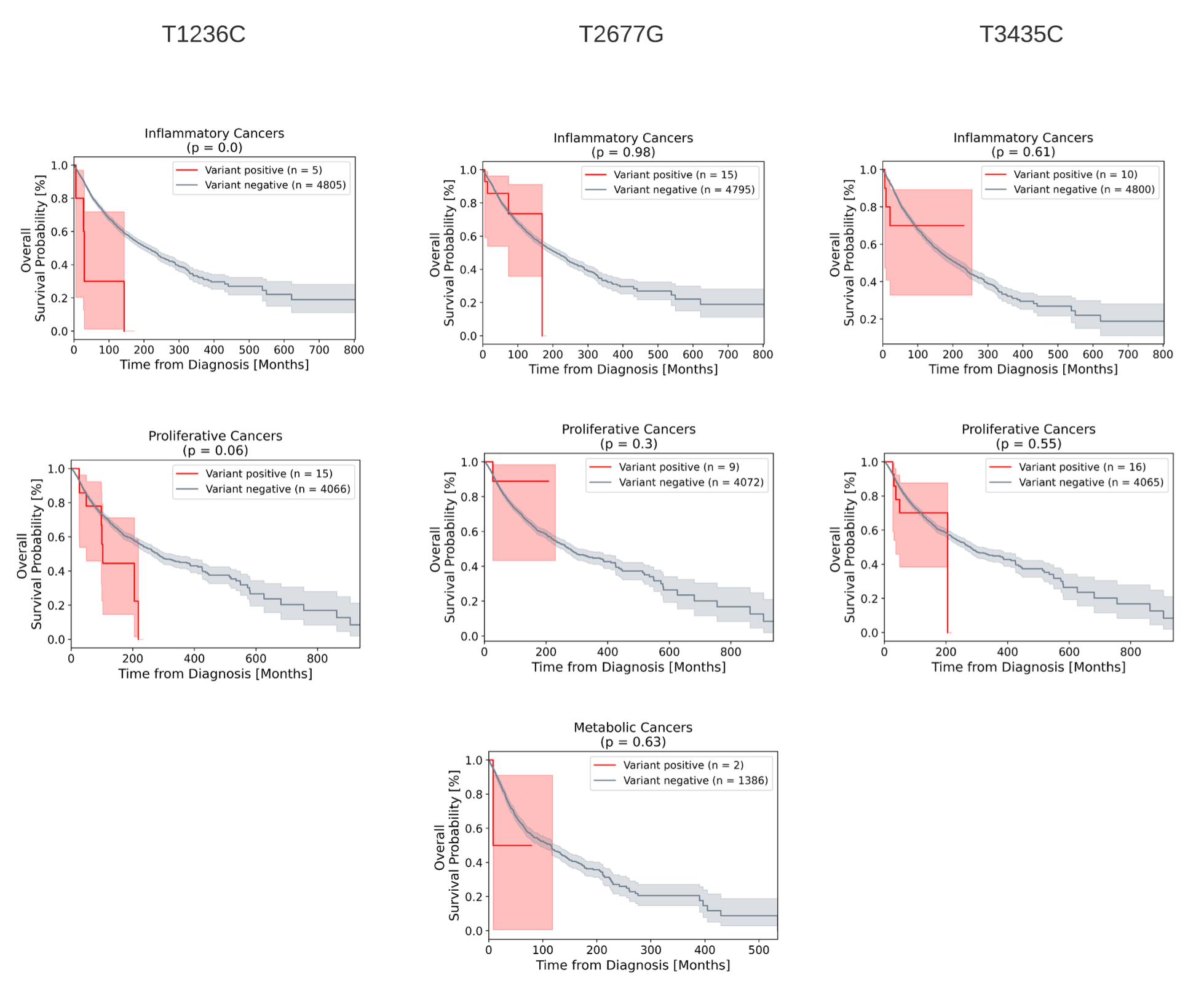

Supplement: S5 Fig — Top row–inflammatory cancers. Middle row- proliferative cancers. Bottom row- metabolic cancers. Categories are described in S1 Table. (PNG) [file pcbi.1012685.s005.png]

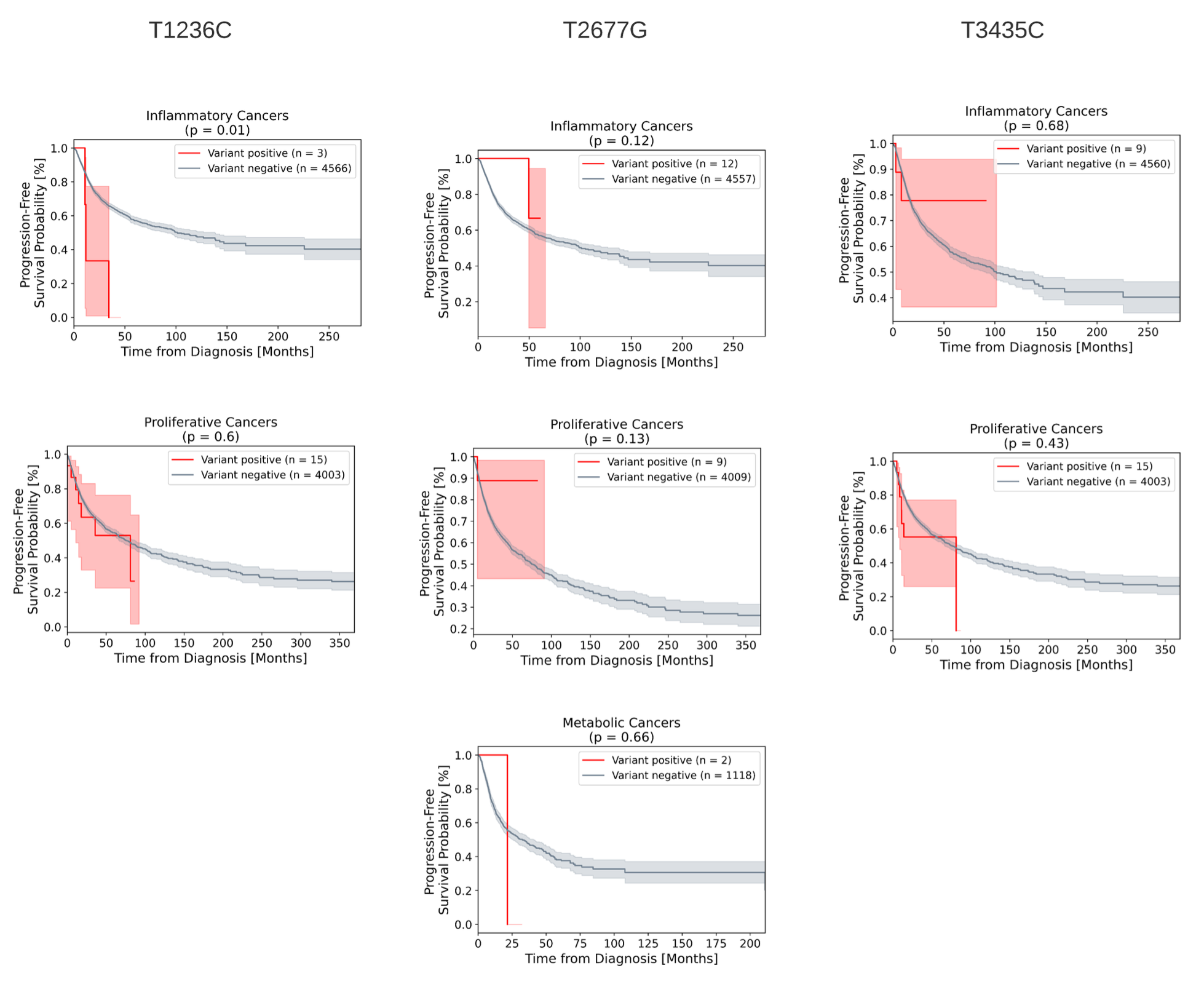

Supplement: S6 Fig — Top row–inflammatory cancers. Middle row- proliferative cancers. Bottom row- metabolic cancers. Categories are described in S1 Table. (PNG) [file pcbi.1012685.s006.png]

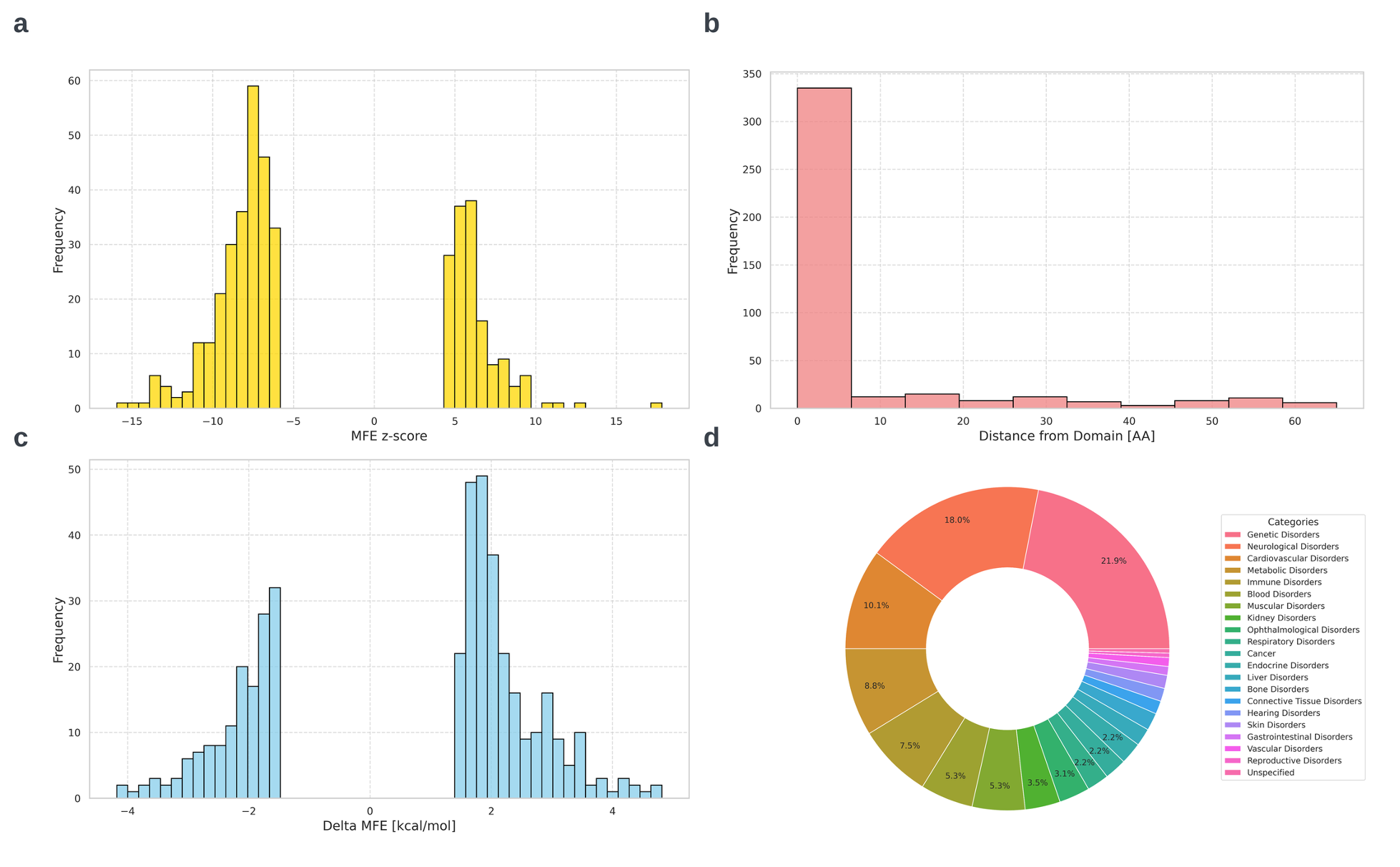

Supplement: S7 Fig — a) Distribution of MFE z-scores in the positions where the variants reside. b) Distribution of variants’ distance from a structural domain c)Distribution of the change in MFE caused by the variants. d) Distribution of disease categories associated with ClinVar variants potentially modifying CTF. Variants not associated with any condition or disease were excluded. (PNG) [file pcbi.1012685.s007.png]

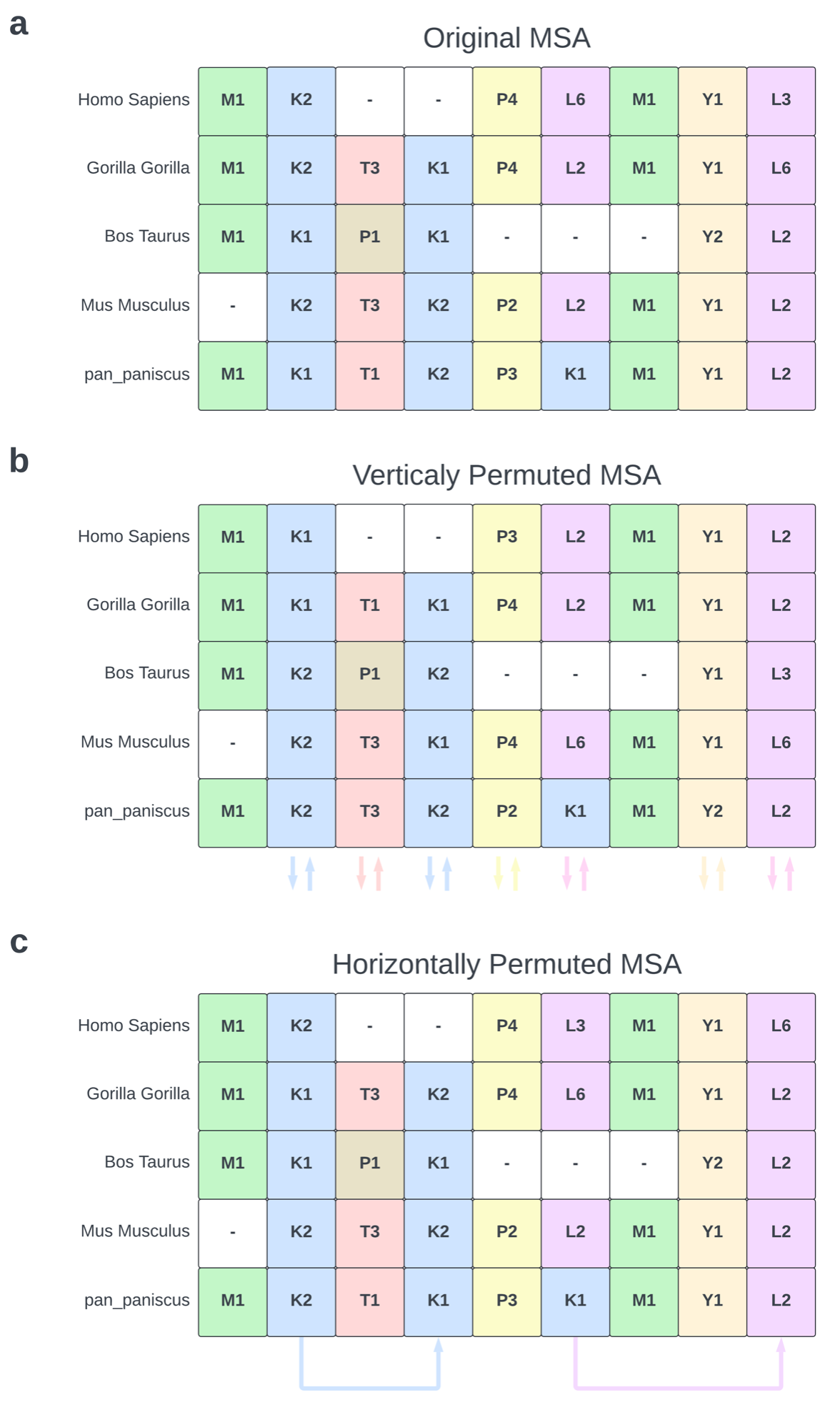

Supplement: S8 Fig — a) Original MSA of five orthologous sequences and amino acids. Each color represents a different amino acid. Synonymous codons are represented by numbering- For example, K1 and K2 are the two synonymous codons that encode for K. lines represent gaps in the MSA. b) Vertically permuted MSA. We shuffled the synonymous codons within each column in the original MSA that had a dominant amino acid. c) Horizontally permuted MSA. We randomly choose pairs of columns with the same dominant amino-acid and swap between the synonymous codons of these columns, keeping them in the same row. (PNG) [file pcbi.1012685.s008.png]

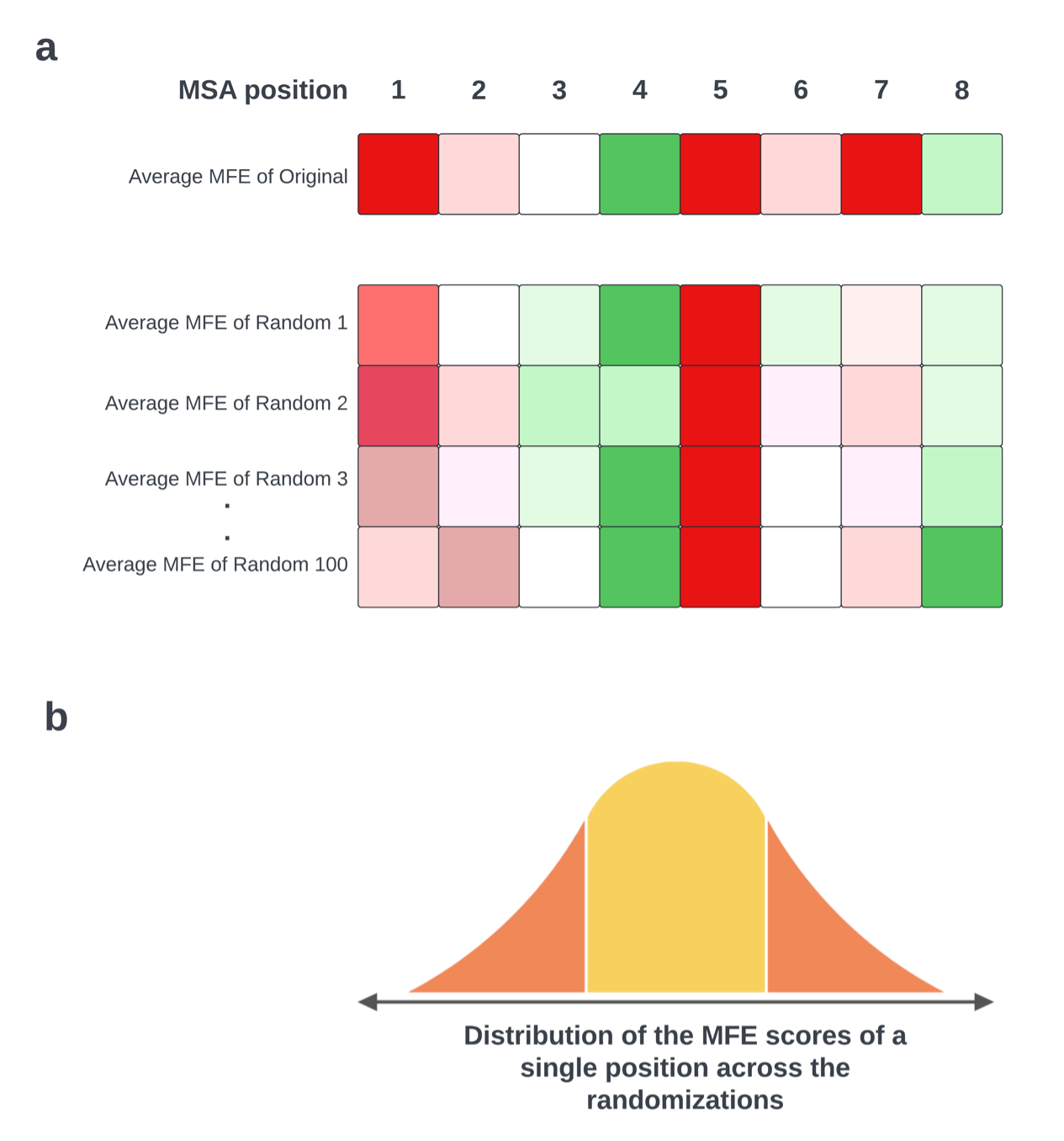

Supplement: S9 Fig — a) An illustration of the MFE scores mapped to the original (top) and randomized (bottom) MSAs. Red hues illustrated strong folding (more negative MFE scores) and green hues illustrates weak folding (less negative MFE scores). b) Taking the MFE scores of all randomizations for a single MSA position enables that calculation of the mean and standard deviation of the random MFE, which is then used to calculate a z-score for the position. (PNG) [file pcbi.1012685.s009.png]
