# Supplementary material for: Computational Analysis of MDR1 Variants Predicts Effect on Cancer Cells via their Effect on mRNA Folding
Source: PLoS Comput Biol. 2024 Dec 26;20(12):e1012685. doi: 10.1371/journal.pcbi.1012685 (PMC11670953; doi:10.1371/journal.pcbi.1012685)
Supplement: S1 Table — (DOCX) [file pcbi.1012685.s010.docx]

| Cluster | Cancer Types |
| --- | --- |
| Metabolic Cancers | LAML, UCS, HNSC, ESCA, UVM, CHOL, LIHC |
| Proliferative Cancers | BLCA, SKCM, SARC, COAD, UCEC, MESO, ACC, LUAD, KIRC, KIRP, DLBC, TGCT, PRAD |
| Inflammatory Cancers | PAAD, LGG, CESC, GBM, READ, LUSC, BRCA, STAD, THCA, OV, KICH, PCPG, THYM |
