# Supplementary material for: Computational Analysis of MDR1 Variants Predicts Effect on Cancer Cells via their Effect on mRNA Folding
Source: PLoS Comput Biol. 2024 Dec 26;20(12):e1012685. doi: 10.1371/journal.pcbi.1012685 (PMC11670953; doi:10.1371/journal.pcbi.1012685)
Supplement: S2 Table — Each cell displays the MDR1 fold change and the corresponding p-value in parenthesis. (DOCX) [file pcbi.1012685.s011.docx]

| Cancer Clusters \ Variants | T1236C | T2677G | T3435C |
| --- | --- | --- | --- |
| Metabolic | - | 2.86 (0.09) | - |
| Proliferative | 1.37 (0.22) | 0.82 (0.46) | 0.83 (0.49) |
| Inflammatory | 0.98 (0.36) | 1.53 (0.11) | 1.17 (0.28) |
