# Supplementary material for: Computational Analysis of MDR1 Variants Predicts Effect on Cancer Cells via their Effect on mRNA Folding
Source: PLoS Comput Biol. 2024 Dec 26;20(12):e1012685. doi: 10.1371/journal.pcbi.1012685 (PMC11670953; doi:10.1371/journal.pcbi.1012685)
Supplement: S1 Text — (DOCX) [file pcbi.1012685.s016.docx]

**Methods for the co-translational folding model**

Obtaining orthologs of MDR1

We Downloaded all available orthologous sequences of the MDR1 coding region from ENSEMBL[1]. We downloaded both amino-acid sequences and nucleotide sequences.

There was a total of 149 orthologues sequences, obtained from 118 unique organisms. This is the full list of organisms-

'aquila_chrysaetos_chrysaetos', 'balaenoptera_musculus',

'bison_bison_bison', 'bos_indicus_hybrid', 'bos_mutus',

'bos_taurus', 'caenorhabditis_elegans', 'camelus_dromedarius',

'canis_lupus_dingo', 'canis_lupus_familiaris', 'capra_hircus',

'carassius_auratus', 'carlito_syrichta', 'catagonus_wagneri',

'cavia_porcellus', 'cebus_capucinus', 'cervus_hanglu_yarkandensis',

'chinchilla_lanigera', 'chlorocebus_sabaeus',

'choloepus_hoffmanni', 'coturnix_japonica',

'cricetulus_griseus_chok1gshd', 'crocodylus_porosus',

'cyclopterus_lumpus', 'dasypus_novemcinctus',

'delphinapterus_leucas', 'dipodomys_ordii', 'echinops_telfairi',

'electrophorus_electricus', 'eptatretus_burgeri',

'equus_asinus_asinus', 'equus_caballus', 'erinaceus_europaeus',

'erpetoichthys_calabaricus', 'ficedula_albicollis',

'fundulus_heteroclitus', 'gallus_gallus', 'geospiza_fortis',

'gorilla_gorilla', 'heterocephalus_glaber_female',

'ictidomys_tridecemlineatus', 'jaculus_jaculus',

'loxodonta_africana', 'mandrillus_leucophaeus',

'marmota_marmota_marmota', 'meleagris_gallopavo',

'mesocricetus_auratus', 'microcebus_murinus',

'microtus_ochrogaster', 'monodelphis_domestica',

'monodon_monoceros', 'moschus_moschiferus', 'mus_caroli',

'mus_musculus', 'mus_pahari', 'mus_spicilegus', 'mus_spretus',

'mustela_putorius_furo', 'myotis_lucifugus', 'nannospalax_galili',

'neovison_vison', 'nomascus_leucogenys', 'notamacropus_eugenii',

'notechis_scutatus', 'ochotona_princeps', 'octodon_degus',

'oncorhynchus_kisutch', 'oncorhynchus_tshawytscha',

'oryctolagus_cuniculus', 'otolemur_garnettii',

'ovis_aries_rambouillet', 'pan_paniscus', 'pan_troglodytes',

'panthera_leo', 'panthera_pardus', 'panthera_tigris_altaica',

'parus_major', 'peromyscus_maniculatus_bairdii',

'petromyzon_marinus', 'phascolarctos_cinereus', 'phocoena_sinus',

'physeter_catodon', 'poecilia_formosa', 'poecilia_latipinna',

'poecilia_reticulata', 'pongo_abelii', 'procavia_capensis',

'prolemur_simus', 'propithecus_coquereli', 'pteropus_vampyrus',

'pygocentrus_nattereri', 'rattus_norvegicus',

'rhinolophus_ferrumequinum', 'saccharomyces_cerevisiae',

'salmo_salar', 'salmo_trutta', 'sarcophilus_harrisii',

'sciurus_vulgaris', 'scleropages_formosus', 'serinus_canaria',

'sinocyclocheilus_grahami', 'sorex_araneus', 'sphenodon_punctatus',

'strigops_habroptila', 'struthio_camelus_australis', 'sus_scrofa',

'taeniopygia_guttata', 'terrapene_carolina_triunguis',

'tupaia_belangeri', 'tursiops_truncatus', 'urocitellus_parryii',

'ursus_americanus', 'ursus_maritimus', 'vicugna_pacos',

'vombatus_ursinus', 'vulpes_vulpes', 'xenopus_tropicalis',

'xiphophorus_maculatus'

Creating a multiple sequence alignment (MSA) from all orthologous sequences

We used Clustal Omega[2] to create an MSA from the orthologous sequences. We then used PAL2NAL[3] and our nucleotide orthologous sequences to convert the protein alignment to a codon alignment.

Creating randomizations of the MSA

**vertical randomizations**

In this version of randomized MSAs we permute synonymous codons within each column. First, we choose to permute only columns with a dominant amino acid, this means that at least 50% of the amino acids in that column are the same one.

Then, we shuffle between the synonymous codons of the dominant amino acid in this column. For example, let’s examine the second column in the MSA illustrated in S8a Fig – the dominant amino acid in this column is Lysine (K), which has two synonymous codons (K1 and K2). In the vertical randomization (S8b Fig) we randomly shuffle these synonymous codons, such that the amino acid MSA stays the same, but the nucleotide MSA is changed. Same was done for all the columns in this illustrated MSA, except columns 1 and 7 which contains the amino-acid M which is encoded by only one codon. We create 100 such randomizations.

**horizontal randomizations**

In this version of randomized MSAs we swap between synonymous codons of two columns with the same dominant amino acid. The definition for a column with a dominant amino acid is the same as the one used for vertical randomizations. We iterate through all amino acids. For each amino acid, for example K, we find all columns for which K is the dominant amino acid. Then, we randomly choose a pair of columns within this set of columns. We swap between the synonymous codons that code for K between these columns, keeping them in the same row. For example, in S8c Fig we see that the second and fourth columns of the illustrated MSA were chosen as a pair. The codons that code for K were swapped between the second and fourth codon positions, within each organism’s sequence. Same was done for the sixth and nineth columns for the L amino acid.

For each amino acid, we randomly choose (protein_length * 10) pairs of columns to shuffle between. This large number of shuffles asserts that we are indeed creating a randomized MSA. We create 100 such randomizations.

Calculating the minimum free energy (MFE) for the original and randomized MSAs

**Calculating per-position MFE scores**

This is an example of a single sequence-

ATGACCATAACGGTC

We use Vienna RNA[4] to calculate the MFE of each position in the MSA. For the folding energy to be meaningful, it needs to be calculated for a sequence and not for a single position; therefore, we first calculate a per-window MFE score:

We use a sliding window with a stride of 1 to obtain sub-sequences from our original sequence. Let’s say we are using a window size of 5 on the example sequence. We will get the following sub-sequences-

1: ATGAC

2: TGACC

3: GACCA

4: ACCAT

5: CCATA

6: CATAA

7: ATAAC

8: TAACG

9: AACGG

10: ACGGT

11: CGGTC

For our actual model we use a window size of 39. For each of these sub-sequences we calculate the MFE. For example-

| window | 1 | 2 | 3 | 4 | 5 | 6 | 7 | 8 | 9 | 10 | 11 |
| --- | --- | --- | --- | --- | --- | --- | --- | --- | --- | --- | --- |
| MFE score | -3 | -3.5 | -4 | -4.5 | -4.2 | -3.8 | -2.5 | -2.1 | -1.7 | -1 | -1.3 |

Next, we use the per-window MFE scores to calculate the MFE score per position. The MFE score of each position is the average of all the scores of the windows in which it resides.

For example, position 5 in the sequence (ATGA**C**CATAACGGTC) is in the windows 1-5. Its MFE score will be equal to -3.84 because (-3-3.5-4-4.5-4.2)/5 = -3.84.

We calculate the per-position MFE scores for all the original sequences of the MDR1 orthologs.

We also perform the same calculations for the randomized sequences.

**Mapping the scores to the MSAs**

Next, we map the scores from the sequences to the nucleotide MSAs:

Continuing with our example sequence, these are the per-positions MFE scores-

| position | 1 | 2 | 3 | 4 | 5 | 6 | 7 | 8 | 9 | 10 | 11 | 12 | 13 | 14 | 15 |
| --- | --- | --- | --- | --- | --- | --- | --- | --- | --- | --- | --- | --- | --- | --- | --- |
| MFE score | -3 | -3.25 | -3.5 | -3.75 | -3.84 | -4 | -3.8 | -3.42 | -2.86 | -2.21 | -1.72 | -1.52 | -1.33 | -1.15 | -1.3 |

And let’s say that the respective row of this sequence in the MSA is this one-

ATGACCATA---ACG---GTC

Then the scores will be mapped accordingly, considering the positions of the gaps.

After we perform this for all sequences, we get matrices that are the MFE scores for the MSAs, for example-

| -3 | -3.25 | NaN | -4 | -2.3 |
| --- | --- | --- | --- | --- |
| -4.1 | -1.3 | NaN | -2 | -1 |
| -7 | -2 | -5 | -3.2 | NaN |
| -10 | -4 | -4.5 | -2 | -3 |

Were NaNs are placed in positions of gaps in the MSA.

**Obtaining average MFE per positions**

Next, we average each column, meaning that we obtain the per-position average MFE score across all orthologs.

This score enables us to understand which positions have a signal of strong folding or weak folding across different organisms.

At this point we get a single vector of average per-position MFE scores for the original MSA, 100 such vectors for the vertical randomizations and 100 such vectors for the horizontal MSAs (see S9a Fig).

Calculating z-scores and p-values

Finally, we compare the per-position MFE scores of the original sequence to the random sequences, in order to understand if the signal of low/high MFE is selected for and not simply random due to other constraints in the sequence.

For each position we calculate the mean and standard deviation of MFE scores of the randomizations (S9b Fig), and then we calculate a z-score using the following equation-

$$z_{i}=\frac{{MFE}_{i}- \mu_{i}}{\sigma_{i}}(2)$$

Where ${MFE}_{i}$ is the MFE score of position i in the original sequence and $\mu_{i}$, $\sigma_{i}$ are the mean and standard deviations of the MFE scores of position i in all the randomizations

We can see for example that position 5 in S9a Fig is very strongly folded, but so are the random sequences in this position, which means that this signal is not selected for. On the other hand, position 1 in the original sequence is folded much stronger than the randomizations, so its z-score will be more extreme.

We also compute a p-value for each position using the z-scores. Then we perform an FDR correction for the p-values of all positions in the sequence.

Defining positions with significantly conserved low/high MFE

We define positions with significantly low MFE as positions with z<0 and corrected_p_value < 0.1 when comparing to both kinds of randomizations.

Similarly, we define positions with significantly high MFE as positions with z>0 and corrected_p_value < 0.1 when comparing to both kinds of randomizations.

**References**

[1] F. Cunningham *et al.*, “Ensembl 2022,” *Nucleic Acids Res*, vol. 50, no. D1, pp. D988–D995, Jan. 2022, doi: 10.1093/nar/gkab1049.

[2] F. Sievers *et al.*, “Fast, scalable generation of high-quality protein multiple sequence alignments using Clustal Omega,” *Mol Syst Biol*, vol. 7, p. 539, Oct. 2011, doi: 10.1038/msb.2011.75.

[3] M. Suyama, D. Torrents, and P. Bork, “Suyama M, Torrents D, Bork P.. PAL2NAL: robust conversion of protein sequence alignments into the corresponding codon alignments. Nucleic Acids Res 34: W609-W612,” *Nucleic Acids Res*, vol. 34, pp. W609-12, Aug. 2006, doi: 10.1093/nar/gkl315.

[4] R. Lorenz *et al.*, “ViennaRNA package 2.0,” *Algorithms Mol Biol*, vol. 6, p. 26, Nov. 2011, doi: 10.1186/1748-7188-6-26.
