# Supplementary material for: Computational Analysis of MDR1 Variants Predicts Effect on Cancer Cells via their Effect on mRNA Folding
Source: PLoS Comput Biol. 2024 Dec 26;20(12):e1012685. doi: 10.1371/journal.pcbi.1012685 (PMC11670953; doi:10.1371/journal.pcbi.1012685)
Supplement: Attachment — Submitted filename: Fung. Canc. Res.nihms763437.pdf [file pcbi.1012685.s017.pdf]

Author Manuscript
Author Manuscript
Author Manuscript
Author Manuscript

HHS Public Access
Author manuscript
Cancer Res. Author manuscript; available in PMC 2016 March 09.

Published in final edited form as:
Cancer Res. 2014 January 15; 74(2): 598–608. doi:10.1158/0008-5472.CAN-13-2064.

MDR1 Synonymous Polymorphisms Alter Transporter Specificity and Protein Stability in a Stable Epithelial Monolayer

King Leung Fung[1], **James Pan**[1], **Shinobu Ohnuma**[1], **Paul E. Lund**[1], **Jessica N. Pixley**[1], **Chava Kimchi-Sarfaty**[2], **Suresh V. Ambudkar**[1], and **Michael M. Gottesman**[1]

[1]Laboratory of Cell Biology, National Cancer Institute, Center for Cancer Research, National Institutes of Health, Bethesda, MD, USA

[2]Center for Biologics Evaluation and Research, Division of Hematology, Food and Drug Administration, Bethesda, MD, USA

Abstract

The drug efflux function of P-glycoprotein (P-gp) encoded by *MDR1* can be influenced by genetic polymorphisms, including two synonymous changes in the coding region of *MDR1*. Here we report that the conformation of P-gp and its drug efflux activity can be altered by synonymous polymorphisms in stable epithelial monolayers expressing P-gp. Several cell lines with similar *MDR1* DNA copy number were developed and termed LLC-MDR1-WT (expresses wild-type P-gp), LLC-MDR1-3H (expresses common haplotype P-gp), and LLC-MDR1-3HA (a mutant that carries a different valine codon in position 3435). These cell lines express similar levels of recombinant mRNA and protein. P-gp in each case is localized on the apical surface of polarized cells. However, the haplotype and its mutant P-gps fold differently from the wild-type, as determined by UIC2 antibody shift assays and limited proteolysis assays. Surface biotinylation experiments suggest that the non-wild-type P-gps have longer recycling times. Drug transport assays show that wild-type and haplotype P-gp respond differently to P-gp inhibitors that block efflux of rhodamine-123 or mitoxantrone. In addition, cytotoxicity assays show that the LLC-MDR1-3H cells are more resistant to mitoxantrone than the LLC-MDR1-WT cells after being treated with a P-gp inhibitor. Expression of polymorphic P-gp, however, does not affect the host cell's morphology, growth rate, or monolayer formation. Also, ATPase activity assays indicate that neither basal nor drug-stimulated ATPase activities are affected in the variant P-gps. Taken together, our findings indicate that "silent" polymorphisms significantly change P-gp function, which would be expected to affect interindividual drug disposition and response.

Keywords

LLC-PK1; pharmacogenomics; ABC transporter; ABCB1; MDR1; polymorphism; multidrug resistance; P-glycoprotein

---

Corresponding author: Michael M. Gottesman, Laboratory of Cell Biology, Center for Cancer Research, National Cancer Institute, National Institutes of Health, Bethesda, MD, USA 20892. Tel: +1 (301) 496 1921, Fax: +1 (301) 402 0450. mgottesman@nih.gov.
Current address for J. Pan: Stanford University Medical School, Stanford, CA. For S. Ohnuma: Dept. of Surgery, Tohoku University Hospital, Sendai, Japan. For P. Lund: University of Michigan, Ann Arbor, MI.

Disclosure of Potential Conflicts of Interest
No potential conflicts of interest were disclosed.

Author Manuscript Author Manuscript Author Manuscript Author Manuscript

Introduction

MDR1 (P-glycoprotein [P-gp], ABCB1) is one of the major drug transporters found in humans. This gene encodes P-gp, an efflux transporter in the plasma membrane that actively transports a broad range of drugs in an ATP-dependent manner (1). It is found in multiple organs (2), and is expressed in the trophoblast layer of the placenta during pregnancy (3). Mice carrying null *abcb1a* and *abcb1b* genes are viable, but have altered pharmacokinetics of many drugs that are P-gp substrates (4–6). American collies carrying truncated *MDR1* genes have lower tolerance to vincristine and the deworming agent ivermectin, a substrate of P-gp (7, 8). Overexpression of P-gp is a common cause of acquired drug resistance in cultured cancer cells (9–13). In polarized epithelia, P-gp is located on the apical membrane, facilitating transport in a directional manner (14, 15).

P-gp contains two important functional domains: the substrate binding site, and the ATPase domain. It is well documented that mutations in these domains change P-gp function (reviewed in (16, 17)). In humans, the *MDR1* gene is highly polymorphic, with at least 50 coding single nucleotide polymorphisms (SNPs) in the *MDR1* coding region documented. In particular, three SNPs at positions 1236C>T, 2677G>T and 3435C>T, which form the most common haplotype, have been studied extensively (16, 18–20). Since the first report showing the alteration of P-gp function with these SNPs (18), many studies have been done to define the influence of these SNPs individually, or of the complete haplotype. However, the results of these population-based studies are indecisive, possibly due to variations in terms of experimental settings including inadequate population sizes to assure statistical significance, incomplete sequence of individuals, differences in tissue-specific P-gp expression, and other unknown environmental factors (21).

The synonymous SNP 3435C>T, usually part of the haplotype noted above, plays an influential role in P-gp function, including elevated digoxin, cyclosporin A (CsA), and fexofenadine bioavailability (22–24). Our previous study using a vaccinia virus-based transient expression system showed that wild-type P-gp and its haplotype are different in function (25). We also suggested that differences in protein characteristics of 3435C>T, such as those mentioned above, might be related to the introduction of a rare codon that alters the translational rhythm and folding of P-gp. However, there are technical limitations in vaccinia virus-based high-level transient expression systems that led us to conduct transport studies and protein stability experiments in polarized cells. To study haplotype P-gp and compare its function with wild-type P-gp under conditions more physiological than those in the transient expression experiments, we developed stable cell lines in which the human *MDR1* gene and its variants were translated from recombinant DNA and inserted into genomic DNA in a subclone of LLC-PK1 cells that can form polarized monolayers.

Materials and Methods

Cell culture and materials

The LLC-PK1 cell line was obtained from American Type Culture Collection, and maintained in Medium199 + 3% (v/v) Fetal Bovine Serum (FBS) + 1% penicillin/

Author Manuscript Author Manuscript Author Manuscript Author Manuscript

streptomycin. The recombinant cell lines were incubated in the same medium with 500 μg/ml geneticin. KB-3-1, KB-V1 and KB-8-5 cells were cultured in DMEM medium + 10% FBS and 1% penicillin/streptomycin. Cells were cultured at 37 °C with 5% CO_2 and relative humidity maintained at 95%.

Cell culture media and geneticin were purchased from Invitrogen. Biotin, paraformaldehyde, verapamil, vinblastine, rodamine-123, calcein-AM, mitoxantrone, trypsin, soybean trypsin inhibitor, 3-(4,5-dimethylthiazol-2-yl)-2,5-diphenyltetrazolium bromide (MTT) and valinomycin were obtained from Sigma. Bodipy-FL–vinblastine was obtained from Molecular Probes. Restriction enzymes were obtained from New England Biolabs. The antibodies were purchased from the following companies: DAKO (C219, MRK16); Invitrogen (IgG2a-Alexa 488, CY™3-Streptavidine); eBiosciences (UIC2-PE, 17F9, IgG2a-HRP; Strepavidin-PE) and Jackson Immuno Research (IgG2a-FITC). ECL reagents were obtained from GE Healthcare. ^{125}I-iodoarylazidoprazosin (2200 Ci/mmole) was obtained from PerkinElmer Life Sciences.

Preparation of pcDNA-MDR1 constructs

Details concerning the preparation of constructs can be found in Supplementary Materials and Methods.

Generation of LLC-MDR1 cell lines

Generation of cell lines is detailed in Supplementary Materials and Methods.

Protein extraction and immunoblot analysis

Total protein extraction from cell culture and protein concentration estimation methods were reported previously (26). For SDS-PAGE, protein samples (50 μg) were loaded onto a 3–8% Tris-acetate gel (Invitrogen). Separated proteins were transferred to a nitrocellulose membrane by iBlot (Invitrogen). The membrane was blocked in PBS + 20% milk and incubated overnight with C219 antibody (1:2000) followed by incubation with anti IgG-2a-HRP antibody and ECL+ (GE Healthcare).

Southern blot analysis

Total genomic DNA was extracted using a GenElute Mammalian Genomic DNA Purification Kit (Sigma). Total DNA (50 μg) and pcDNA-MDR1(1236C-2677G-3435C) (as copy number standard) were digested with NdeI for 24 hrs and separated in a 0.7% agarose gel. DNA in the gel was denatured and was transferred onto a Hybond-XL membrane (GE Healthcare). The membrane was washed briefly with 2× saline-sodium citrate and soaked with Hybridsol in 42 °C for 2 hrs. A NdeI-digested DNA [^{32}P]-labeled DNA probe was prepared by Ready-To-Go DNA Labeling kit (GE Healthcare) and was added on the membrane for 16 hour incubation at 42 °C. The membrane was washed with 2 × SSC/0.1%SDS at 65 °C for 30 min, 2 × 0.1 × SSC/0.1% SDS at 65 °C for 10 min. The image was recorded by a phosphoimager and was analyzed by ImageQuant TL (GE Healthcare).

Author Manuscript Author Manuscript Author Manuscript Author Manuscript

TaqMan qRT-PCR

Expression levels of human *MDR1* genes were measured using the TaqMan method. 1 µg of total RNA, isolated by the RNeasy mini kit (Qiagen), was used to synthesize cDNA using the High capacity cDNA Reverse Transcription Kit (Invitrogen). cDNA was mixed with TaqMan Universal PCR Master Mix (Invitrogen), run on an ABI Prism 7900HT Sequence Detection System (Invitrogen) as per the manufacturer's instructions. Porcine plasma membrane calcium ATPase 4 gene (PMCA4) was used as the reference gene.

Confocal microscopy

Cells were grown on Transwell 3470 membrane inserts (Corning) until confluent. Tight cell monolayers were fixed by treatment with 4% paraformaldehyde for 10 min, then incubated with PBS + 1 mM $MgCl_2$ + 0.1 mM $CaCl_2$ + 1% BSA for 1 hr. To label P-gp, cell monolayers were incubated with MRK16 (1:100) for 1 hr, followed by incubation with anti-IgG2a-Alexa 488 antibody. To label nuclei, cells were incubated with DAPI (300 nM). For cell surface labeling, biotin was incubated for 15 min before cell monolayers were fixed. Strepavidin-PE (1:100) was added to the fixed cell monolayers for 1 hr. Fluorescence images were acquired with a Zeiss LSM-510 confocal microscope, and the images were analyzed by LSM Image Browser (version 4.2.0) (Zeiss).

Cell growth assay

Cells were seeded on 60 mm culture dishes and cultured at 37 °C. Starting from day 3 after cell seeding, 3 dishes of cells from each cell line were trypsinized. Cell numbers were recorded daily until day 14. Cell counting was measured by the AutoT4 cellometer (Nexcelom Bioscience).

Cell surface immunolabeling by flow cytometry

Cell surface P-gp expression was detected by incubating cells with 1 µg of MRK16, UIC2, and 17F9 antibodies per 200,000 cells for 30 min. For experiments using CsA (10 µM), cells were pre-incubated with drug for 10 min. After primary antibody incubation, cells were incubated with anti-mouse IgG2a FITC-conjugated antibody for 40 min.

Drug influx/efflux assay

Actively growing cells (2×10^5) were harvested by trypsinization and were incubated in IMDM + 5% FBS medium, with rhodamine 123 (0.5 µg/ml), mitoxantrone (5 µM), or bodipy-FL–vinblastine (0.5 µM) in the presence or absence of CsA (10 µM), DCPQ (50 nM), Tariquidar (50 nM), verapamil (5 µM), digoxin (125 µM) at 37 °C. Cells were washed and further incubate with IMDM or IMDM with drugs for 40 min before FACS analysis.

Limited trypsin digestion assay

Limited protein digestions by trypsin were performed with crude membranes. 3 µg of the crude membranes were treated with different concentrations of trypsin for 5 min at 37 °C. The reaction was stopped with 5 × excess of soybean trypsin inhibitor. Remaining P-gp was detected by Western blot analysis using C219 antibody.

Author Manuscript

Cell surface P-gp biotinylation

For cell surface biotinylation, cells were washed with ice cold PBS, and then incubated with 0.2 mg/ml EZ-Link Sulfo-NHS-LC-Biotin (Thermo Scientific) in ice cold PBS for 30 minutes. Excess biotin was washed with glycine/PBS and PBS. For flow cytometry analysis, cells were trypsinized, washed, and incubated with MRK16 + IgG2a-FITC (0.5 μg per 200,000 cells) + CY™3-Streptavidine (1 : 50). Labeling of cells with Sulfo-NHS-LC-Biotin just before trypsinization was considered as time 0. The percentage of biotinylated P-gp remaining at each time point was determined by measuring the percentage of biotinylated P-gp cells remaining compared to the number of biotinylated P-gp cells at time 0. Data calculations were performed by CellQuest software (BD Biosciences).

Cytotoxicity assay

The cytotoxicity of drugs on LLC-MDR1 cells was measured by a colorimetric viability assay using MTT as previously described (26). Cytotoxicity (IC_{50}) was defined as the drug concentration that reduced cell viability to 50% of the untreated control.

Crude membrane preparations and ATPase assays

The crude membranes from cells were prepared as described previously (27). ATPase activities were measured as reported previously (28). Briefly, crude membrane protein (10 μg protein per 100 μl) was incubated at 37 °C for 30 min in 100 μl of reaction buffer (50 mM MOPS-KOH, pH 7.5, 50 mM KCl, 5 mM sodium azide, 1 mM EGTA, 1 mM ouabain, 10 mM $MgCl_2$) in the presence and absence of 0.3 mM sodium orthovanadate (Vi). The reaction was initiated by the addition of 5 mM ATP for 20 min at 37 °C. SDS solution (2.5% v/v) was added to terminate the reaction, and the amount of inorganic phosphate released was quantified with a colorimetric reaction, as described previously (28). The basal P-gp ATPase activity was determined by subtracting the ATPase activity in the P-gp-containing membranes with the ATPase activity in the control membranes in the same experiment. Drug-stimulated P-gp ATPase activity was measured in an ATPase reaction mixture that contained 2.5 μM paclitaxel, 10 μM vinblastine, 10 μM valinomycin and 30 μM verapamil, and the activity obtained was corrected for the basal ATPase activity.

Photoaffinity labeling with ^{125}I-Iodoarylazidoprazosin

Photoaffinity assays were performed by incubating crude membranes (1 mg/ml) with drugs for 10 min at room temperature in 50 mM Tris-HCl, pH 7.5 followed by the addition of 3–6 nM [^{125}I]-IAAP (2200 Ci/mmol). The samples were cross-linked and incorporation of [^{125}I]-IAAP was determined as described previously (29).

Statistical analysis

Statistical significance of the experimental results was obtained by the two-sample t test. Results were considered statistically significant at $P < 0.05$.

Author Manuscript Author Manuscript Author Manuscript Author Manuscript

Results

Characterization of LLC-PK1 cell lines expressing wild-type, haplotype and mutant P-gps

To characterize the effect of P-gp variants, stable cell lines expressing human P-gp were developed. The original LLC-PK1 cell line, isolated from porcine kidney proximal tubule epithelial cells (30), expresses low level of P-gp (31). Taking this into consideration, the LLC-PK1 subclone termed LLC-PK1#7 was prepared by clonal selection. LLC-PK1#7 has undetectable endogenous P-gp and is unable to efflux P-gp substrates including rhodamine-123 and calcein-AM (data not shown).

The *MDR1* wild-type (pTM1-MDR1(1236C-2677G-3435C)), haplotype (pTM1-MDR1(1236T-2677T-3435T)), and a MDR1 haplotype mutant encoding the isoleucine at 1145 using a rarer tRNA (pTM1-MDR1(1236T-2677T-3435A)), were used for this study. After transfection, LLC-PK1 clones were isolated and grown under the same culture conditions. During establishment of stable cells, we did not select P-gp expression with P-gp substrates such as colchicine or vinblastine to prevent endogenous P-gp induction and/or alteration of other endogenous resistance mechanisms (32, 33). We designated the cell lines in this study as follows: LLC-vector (vector-transfected cells), LLC-MDR1-WT (MDR1(1236C-2677G-3435C)-expressing cells), LLC-MDR1-3H (MDR1(1236T-2677T-3435T)-expressing cells) and LLC-MDR1-3HA (MDR1(1236T-2677T-3435A)-expressing cells). As shown in Figure 1A, all the P-gp-expressing cell clones, numbered 1–4, have one copy of *MDR1* cDNA. qRT-PCR analysis determined that the transfected DNA expressed a comparable level of *MDR1* transcripts (Figure 1B, 2–4). Recombinant P-gp expression was identified by Western blotting using C219 antibody. In Figure 1C, P-gp was not detectable in the LLC-vector cells (lane 1), but was evident in the P-gp-expressing cells (lanes 2–4). P-gp in all P-gp-expressing LLC-PK1 cell lines was found as a single band with an apparent molecular weight of ~160 kDa and no immature bands were detected (Figure 1C, Figure S1). The P-gp protein from LLC-MDR1-WT cells had lower mobility than the P-gp expressed in High-five insect cell membranes, indicating that P-gp is likely N-glycosylated in the LLC-PK1 cells (Figure S1). The N-glycosylation pattern of the different P-gps may differ, since we observed that the WT-P-gp (Figure 1C, lane 2) had slightly higher mobility than the 3H-P-gp (Figure 1C, lane 3) and the 3HA-P-gp (Figure 1C, lane 4). P-gp-expressing cell lines expressed high levels of P-gp and all the clones expressed comparable levels. P-gp expression in the KB-V1 cells (Figure 1C, lane 5) was greater than in KB-8-5 cells (Figure 1C, lane 6), as expected.

We compared the growth rate of the LLC-PK1 cell lines. The cells were allowed to grow in culture dishes for two weeks until confluency. The growth curves indicated that these cell lines had similar growth rates (Figure 1D).

To determine whether P-gp polarization could be affected by P-gp polymorphisms and synonymous mutations, cell surface immunolabeling was performed on LLC-PK1 cell monolayers. Cells were grown in Transwell chambers until cell monolayers were formed. Light microscopy images showed no significant morphological differences among the tested cell lines (Figure 1E, upper panel). Using confocal microscopy, in the P-gp-expressing cells, P-gp signals (green) were detected on the apical side, but not in the vector-transfected stable

Author Manuscript

cells (LLC-vector). WT-P-gp, 3H-P-gp and 3HA-P-gp expression on the apical membranes were comparable (Figure 1E, middle panel). On the basolateral side, as expected, P-gp was undetectable (Figure 1E, lower panel). These results indicate that in the LLC-PK1 cells, P-gp polymorphisms do not affect protein expression or targeting to the apical cell surface.

MDR1 mutations alter P-gp conformation in the LLC-PK1 cells

To determine whether polymorphisms of *MDR1* could change protein conformation, we examined the LLC-MDR1 cell lines with the conformation-sensitive antibody UIC2. As shown in Figure 2A, the vector cells showed minimal reactivity towards all antibodies tested (black histogram). The wild-type and haplotype P-gp-expressing cells showed high and comparable reactivity with conformation-insensitive MRK16 and 17F9 antibodies. However, these P-gp-expressing cells showed differential reactivity with UIC2 and with UIC2 in the presence of cyclosporine A (10 μM).

To further examine the effect of conformation differences, the accessibility to various trypsin concentrations of P-gp was determined. Figure 2B shows Western blots of wild-type and polymorphic forms of P-gp with trypsin. Membrane proteins from each P-gp-expressing cell line were prepared and they were digested for 5 min with increasing amounts of trypsin, and the remaining P-gp protein was analyzed using C219 antibody. For each protein sample, the concentration of trypsin required for a 50% decrease in P-gp was determined and plotted. We observed differences in trypsin sensitivity with LLC-MDR1-WT (most sensitive) < LLC-MDR1-3H < LLC-MDR1-3HA. The fold change in IC_{50} for LLC-MDR1-WT and LLC-MDR1-3H was 1.2-fold and 1.4-fold for LLC-MDR1-3HA. In the presence of 50 μM verapamil, a P-gp inhibitor, there was no difference in the IC_{50} values for trypsin digestion. WT-P-gp, 3H-P-gp and 3HA-P-gp were more resistant to trypsin in the presence of verapamil, as we have previously observed using transiently-expressed wild-type and haplotype P-gps (25).

P-gp synonymous polymorphisms influence protein stability

To elucidate the impact of conformation changes of P-gp by its polymorphisms in LLC-PK1 cells, we determined the half-life of plasma membrane P-gp in LLC-PK1 cell lines by cell surface biotinylation. Cells were labeled with biotin for 30 min and cultured for 24, 48 and 72 hrs. Cells were stained with strepavidin-PE and MRK16 antibody. The remaining biotinylated P-gp at each time point was determined by flow cytometry and a plot was drawn to calculate the time required for 50% disappearance of biotinylated P-gp (Figure 2B). The mean half-lives of plasma membrane P-gp were 45 hrs (LLC-MDR1-WT), 47 hrs (LLC-MDR1-3H) and 53 hrs (LLC-MDR1-3HA), respectively (Figure 3), which are significantly different from each other ($p < 0.05$), and consistent with the *in vitro* trypsin sensitivity experiments shown in Figure 3.

Synonymous mutations affect P-gp function

The effect of drug transport function of P-gp wild-type and its variants in LLC-PK1 cells, was tested using rhodamine-123 with various P-gp inhibitors. In Figure 4, FACS histograms show transport of rhodamine 123 by cells transfected with the LLC-vector, LLC-MDR1-WT, LLC-MDR1-3H, and LLC-MDR1-3HA cells. In the absence of P-gp inhibitors, efflux

Author Manuscript Author Manuscript Author Manuscript Author Manuscript

of rhodamine-123 by all P-gp-expressing LLC-PK1 cell lines was the same. Complete inhibition of rhodamine 123 efflux was observed when a high concentration of verapamil (20 μM) was used (Figure S2). Similar observations were also made for high concentrations of the P-gp inhibitors tariquidar (200 nM) and cyclosporine A (20 μM) (data not shown). However, we observed differential inhibition of P-gp function when sub-optimal inhibitor concentrations were used. For example, in the presence of 50 nM tariquidar, efflux of rhodamine 123 was least affected in LLC-MDR1-3H cells. Also, in the presence of 10 μM cyclosporine A or 5 μM verapamil, the LLC-MDR1-3HA cells showed slightly higher efflux of rhodamine 123. When we tested the efflux of rhodamine 123 in the presence of digoxin (125 μM), the LLC-MDR1-3HA cells showed the poorest inhibition to digoxin compared to the LLC-MDR1-3H cells and the LLC-MDR1-WT cells, consistent with the original clinical observations that the P-gp haplotype affected digoxin levels in patients (18). These results suggest that the variant forms of P-gp have different sensitivities to the inhibitors digoxin, CsA, and tariquidar.

The LLC-vector cells were used as a control. This cell line was not able to efflux rhodamine 123, and P-gp inhibitors did not influence its accumulation of rhodamine 123 (Figures 4 and 5, black histogram).

Variant P-gps affect drug efflux and cytotoxicity

The stable LLC-PK1 cells expressing human P-gps enabled us to conduct short-term assays and long-term assays. We confirmed that WT-P-gp, 3H-P-gp and 3HA-P-gp expression levels remain unchanged for at least 96 hrs after geneticin removal (results not shown). Growth assays revealed that all the LLC-PK1 cell lines tested had comparable growth rates (Figure 1D), indicating that these cells are suitable for cytotoxicity experiments. We tested two anticancer drugs that are P-gp substrates (vinblastine, mitoxantrone) in the presence or absence of P-gp inhibitors (DCPQ, CsA). We compared the efflux function of P-gp in different LLC-PK1 cell lines using drug transport influx/efflux assays (short-term) and growth-inhibition assays (long-term) by the MTT method. In Figure 5, the P-gp-expressing cells show different mitoxantrone efflux. The LLC-MDR1-3H cells showed significantly higher efflux than the LLC-MDR1-3HA and the LLC-MDR1-WT cells. This difference correlated with increased mitoxantrone resistance of the LLC-MDR1-3H and LLC-MDR1-3HA cells in the presence of mitoxantrone. The calculated IC_{50} values showed that the LLC-MDR1-3H cells (14.6 ± 1.9 μM) were 10 times more resistant to mitoxantrone ($p < 0.001$) than the LLC-MDR1-WT (1.5 ± 0.3 μM) cells, and the LLC-MDR1-3HA (9.2 ± 2.5 μM) cells were 6.3 fold ($p < 0.001$) more resistant than the wild-type cells (Table 1). Also, the fold differences of the IC_{50} values of LLC-MDR1-WT vs LLC-MDR1-3H, LLC-MDR1-WT vs LLC-MDR1-3HA and LLC-MDR1-3H vs LLC-MDR1-3HA cells were also significant. In the presence of 100 nM DCPQ, resistance to mitoxantrone was reversed in the LLC-MDR1-WT (0.2 ± 0.04 μM) and the LLC-MDR1-3HA 0.8 ± 0.2 μM) cells when compared with LLC-vector cells (0.065 ± 0.015 μM). In the presence of DCPQ, the IC_{50} of the LLC-MDR1-3H cells decreased from 14.6 ± 1.9 μM to 3.2 ± 0.6 μM. However, they are still significantly (49.8 fold, $p < 0.001$) resistant to mitoxantrone compared to the LLC-vector and the LLC-MDR1-WT cells (Figure 5 and Table 1). These experiments suggest that the LLC-MDR1-3H cells are less sensitive to DCPQ.

Author Manuscript Author Manuscript Author Manuscript Author Manuscript

The effect of drug efflux function by P-gp variants was tested using vinblastine. For drug accumulation/efflux assays, bodipy-vinblastine was selected as a substrate. The drug efflux assays showed that all the P-gp-expressing cell lines exported bodipy-vinblastine at the same rate. In the presence of 5 μM CsA, all the P-gp-expressing cells showed reduced efflux of bodipy-vinblastine. Using MTT assays, the P-gp-expressing cells had comparable resistance to vinblastine and no statistical significance was found in IC_{50} values between LLC-MDR1-WT vs LLC-MDR1-3H and LLC-MDR1-3HA cells (Figure 5 and Table 1). In the presence of CsA, the P-gp-expressing cells showed reduced and comparable sensitivity to vinblastine.

Effect of substrates on P-gp wild-type and haplotype ATPase activity

The synonymous SNPs 1236C>T and MDR1 3435C>T are in amino acid codons found in the nucleotide binding domains (34). To examine whether P-gp conformation could change drug-stimulated ATPase activity or photoaffinity labeling with a transport substrate, we performed ATPase activity assays and photoaffinity labeling assays. Using membrane protein isolated from LLC-MDR1-WT cells and LLC-MDR1-3H, the basal rate of ATP hydrolysis could be increased significantly by paclitaxel, vinblastine, valinomycin, and verapamil (Figure 6A). There were no significant differences between the cell lines in basal and drug-stimulated ATPase activities.

Photoaffinity labeling assays with [^{125}I]-IAAP in the presence of tasigna, verapamil, paclitaxel, vinblastine, valinomycin and tariquidar indicated that all tested compounds except verapamil could effectively inhibit [^{125}I]-IAAP binding to P-gp (Figure 6B). No significant differences were found between the two cell lines. To further examine the effect of IAAP binding, experiments showed that the IC_{50} value of the LLC-MDR1-WT cell line was 1.9 μM and that of the LLC-MDR1-3H cell line was 2.2 μM ($p > 0.05$).

Discussion

In this study, we developed four recombinant P-gp-expressing cell lines to elucidate the effect of synonymous polymorphisms and haplotypes of *MDR1*. Until recently, the effect of synonymous mutations has generally been ignored. Nevertheless, mounting evidence strongly suggests these polymorphisms can influence gene function and cellular activity via multiple pathways (35–38). In fact, Lawrie *et al* reported that a significant portion of synonymous mutation sites in *Drosophila melanogaster* are subject to strong purifying selection, and the genes harboring these mutations are related to key developmental pathways (39). In the *MDR1* gene, there are two synonymous polymorphisms that frequently occur in certain human populations (34, 40, 41). Many clinical and *in vitro* studies have suggested that the 3435C>T SNP plays an important role in the function of *MDR1*. However, the functional effects of synonymous mutations in *MDR1* have not been studied to any significant degree except after transient, high-level expression (25). Therefore, we decided to use the LLC-PK1 epithelial cell line, which can form a polarized tight monolayer, to evaluate the effect of *MDR1* synonymous polymorphisms, and the effects of codon usage.

Our results show that synonymous mutations do influence protein stability in intact cells in addition to trypsin sensitivity *in vitro* as previously reported (25). The *MDR1* haplotype causes a change in P-gp conformation, as determined using cell surface labeling by UIC2, a

Author Manuscript Author Manuscript Author Manuscript Author Manuscript

P-gp conformation-sensitive antibody (42). We further showed that the 3435C>A synonymous mutation increases binding of UIC2 MAb, and increases resistance to trypsin. The surface protein biotinylation experiments and FACS assays showed the wild-type P-gp had the shortest half-life (45 hrs), followed by the haplotype (47 hrs) and 3HA mutant (53 hrs). These differences are consistent with the protein stability differences demonstrated earlier using the trypsin-sensitivity assay (25). We hypothesize that the difference in protein half-life may be linked to protein recycling. Kim *et al* (43) have reported that P-gp is recycled through a clathrin-dependent pathway and interacts with the AP-2 adaptor complex. The P-gp variations we are studying might cause a change in protein conformation involving a tight turn structure or a di-leucine motif which interacts with AP-2 adaptor complexes. Also, our functional assays showed that, in certain cases, the haplotype mutants are more resistant to inhibitors since the frequency of haplotype forms of P-gp varies in different human populations (19, 20). These results suggest an evolutionary force driving *MDR1* to alter its ability to interact with drugs and dietary materials through altered protein conformation resulting from the haplotype studied here.

However, common synonymous mutations of *MDR1* did not influence mRNA expression, total protein expression, or translocation to the apical membrane surface. Also, the presence of P-gp on the cell surface did not affect cell growth or formation of a tight polarized monolayer. Our observation does not support some clinical reports suggesting that the haplotype allele often correlates with lower P-gp expression (18, 44, 45).

Our cell lines with stable P-gp expression allowed us to conduct physiologically relevant experiments, including drug efflux assays and cytotoxicity assays. Drug efflux assays suggested that the transport function of P-gp variants is dependent on the drug of choice. Our results indicated that P-gp polymorphisms do not affect rhodamine-123 transport in the presence of verapamil or CsA. In contrast, the LLC-MDR1-3H cell line is less sensitive to tariquidar than LLC-MDR1-WT cells. The LLC-MDR1-3HA cells are more resistant to inhibition by digoxin than the cells carrying the haplotype and the wild-type forms of P-gp. Our results with mitoxantrone indicate that the drug transport function of P-gp is significantly influenced by the synonymous mutations in *MDR1*. Mitoxantrone is an anthracene derivative that is a relatively poor substrate of P-gp. Expression of P-gp is able to reduce mitoxantrone accumulation and therefore increase cytotoxic resistance (46). In this study, we found that P-gp polymorphisms directly affect mitoxantrone efflux and cytotoxicity. The exact cause of these observations is unclear, but we hypothesize that the conformational differences affect the relative efficiency of substrate and inhibitor binding between wild-type and haplotype-expressing cells.

In this study, the *MDR*1 1236C>T and 3435C>T SNPs showed a significant impact on the overall folding of P-gp, and not just a localized effect. These “silent” mutations encode glycine 412 and isoleucine 1145, which are found in the first and second ATP-binding domains and are unchanged in the wild-type and haplotype P-gp. These SNPs do not affect basal and drug-stimulated ATPase activity, suggesting changes in conformation in these polymorphic P-gps do not affect ATP binding or hydrolysis (47). These “silent” polymorphisms do not result in mRNA degradation, nor do the resulting P-gp conformational changes result in obstruction in the protein folding pathway (like CFTR),

mis-localization, post-translational modification defects, protein truncation and/or misfolding. In fact, P-gp encoded by the *MDR1* common haplotype is expressed properly on the cell surface, targeted to the apical side in polarized monolayers, and functions as a drug transporter. Furthermore, some of our results indicate that the function of haplotype P-gp might be more efficient than the wild-type P-gp. These results in stably-transfected cells strongly suggest that human P-gp exists in at least two conformational states of equivalent stability. The haplotype form folds differently than wild-type P-gp (48). It seems that the silent polymorphisms encode a protein with alternate (or new) energy minima, similar to the model suggested by Tsai *et al* (48). However, the results do not demonstrate the mechanism of the effect of synonymous mutations on *MDR1*. More experiments are warranted to examine various hypotheses.

In summary, the role of P-gp synonymous polymorphisms was examined in polarized epithelial cells. We showed that the synonymous mutations do influence protein folding and function in stable cells expressing recombinant P-gp. P-gp conformational alterations subtly changed drug efflux function and interaction with P-gp inhibitors. By comparing data from short-term transport assays and cytotoxicity assays, these subtle changes significantly alter cellular cytotoxicity of drugs such as mitoxantrone that do not bind tightly to P-gp. The P-gp-expressing cell lines will help to identify substrates and inhibitors that are influenced by the common variants of P-gp, allowing better predictions about the pharmacokinetics of drugs that are substrates for P-gp, and improved design of clinical studies that seek to inhibit the function of P-gp to reverse drug resistance.

Supplementary Material

Refer to Web version on PubMed Central for supplementary material.

Acknowledgments

This research was supported by the Intramural Research Program of the National Institutes of Health, National Cancer Institute. We thank George Leiman for editorial assistance.

Abbreviations

**ABC**	ATP-binding cassette
**ATP**	adenosine triphosphate
MDR1	multidrug resistant 1 gene (*ABCB*1)
**SNP**	single nucleotide polymorphism
**MTT**	3-(4,5-dimethylthiazol-2-yl)-2,5-diphenyltetrazolium bromide
**DAPI**	4′,6-diamidino-2-phenylindole
**DCPQ**	(2R)-anti-5-(3-[4-(10,11-dichloromethanodibenzo-suber-5-yl)piperazin-1-yl]-2-hydroxypropoxy)quinoline trihydrochloride

Author Manuscript Author Manuscript Author Manuscript Author Manuscript

Author Manuscript Author Manuscript Author Manuscript Author Manuscript

References

1. Ambudkar SV, Lelong IH, Zhang J, Cardarelli CO, Gottesman MM, Pastan I. Partial purification and reconstitution of the human multidrug-resistance pump: characterization of the drug-stimulatable ATP hydrolysis. Proc Natl Acad Sci U S A. 1992; 89:8472–6. [PubMed: 1356264]
2. Fojo AT, Ueda K, Slamon DJ, Poplack DG, Gottesman MM, Pastan I. Expression of a multidrug-resistance gene in human tumors and tissues. Proc Natl Acad Sci U S A. 1987; 84:265–9. [PubMed: 2432605]
3. Mason CW, Buhimschi IA, Buhimschi CS, Dong Y, Weiner CP, Swaan PW. ATP-binding cassette transporter expression in human placenta as a function of pregnancy condition. Drug Metab Dispos. 2011; 39:1000–7. [PubMed: 21430233]
4. Schinkel AH. The physiological function of drug-transporting P-glycoproteins. Semin Cancer Biol. 1997; 8:161–70. [PubMed: 9441946]
5. Schinkel AH, Smit JJ, van Tellingen O, Beijnen JH, Wagenaar E, van Deemter L, et al. Disruption of the mouse mdr1a P-glycoprotein gene leads to a deficiency in the blood-brain barrier and to increased sensitivity to drugs. Cell. 1994; 77:491–502. [PubMed: 7910522]
6. Smit JW, Schinkel AH, Weert B, Meijer DK. Hepatobiliary and intestinal clearance of amphiphilic cationic drugs in mice in which both mdr1a and mdr1b genes have been disrupted. Br J Pharmacol. 1998; 124:416–24. [PubMed: 9641561]
7. Krugman L, Bryan JN, Mealey KL, Chen A. Vincristine-induced central neurotoxicity in a collie homozygous for the ABCB1Delta mutation. J Small Anim Pract. 2012; 53:185–7. [PubMed: 22122243]
8. Roulet A, Puel O, Gesta S, Lepage JF, Drag M, Soll M, et al. MDR1-deficient genotype in Collie dogs hypersensitive to the P-glycoprotein substrate ivermectin. Eur J Pharmacol. 2003; 460:85–91. [PubMed: 12559367]
9. Roninson IB, Chin JE, Choi KG, Gros P, Housman DE, Fojo A, et al. Isolation of human mdr DNA sequences amplified in multidrug-resistant KB carcinoma cells. Proc Natl Acad Sci U S A. 1986; 83:4538–42. [PubMed: 3459187]
10. Shen DW, Fojo A, Chin JE, Roninson IB, Richert N, Pastan I, et al. Human multidrug-resistant cell lines: increased mdr1 expression can precede gene amplification. Science. 1986; 232:643–5. [PubMed: 3457471]
11. Akiyama S, Fojo A, Hanover JA, Pastan I, Gottesman MM. Isolation and genetic characterization of human KB cell lines resistant to multiple drugs. Somat Cell Mol Genet. 1985; 11:117–26. [PubMed: 3856953]
12. Aleman C, Annereau JP, Liang XJ, Cardarelli CO, Taylor B, Yin JJ, et al. P-glycoprotein, expressed in multidrug resistant cells, is not responsible for alterations in membrane fluidity or membrane potential. Cancer Res. 2003; 63:3084–91. [PubMed: 12810633]
13. Bech-Hansen NT, Till JE, Ling V. Pleiotropic phenotype of colchicine-resistant CHO cells: cross-resistance and collateral sensitivity. J Cell Physiol. 1976; 88:23–31. [PubMed: 57118]
14. Horio M, Chin KV, Currier SJ, Goldenberg S, Williams C, Pastan I, et al. Transepithelial transport of drugs by the multidrug transporter in cultured Madin-Darby canine kidney cell epithelia. J Biol Chem. 1989; 264:14880–4. [PubMed: 2570070]
15. Thiebaut F, Tsuruo T, Hamada H, Gottesman MM, Pastan I, Willingham MC. Cellular localization of the multidrug-resistance gene product P-glycoprotein in normal human tissues. Proc Natl Acad Sci U S A. 1987; 84:7735–8. [PubMed: 2444983]
16. Ieiri I. Functional significance of genetic polymorphisms in P-glycoprotein (MDR1, ABCB1) and breast cancer resistance protein (BCRP, ABCG2). Drug Metab Pharmacokinetics. 2012; 27:85–105.
17. Ambudkar SV, Kimchi-Sarfaty C, Sauna ZE, Gottesman MM. P-glycoprotein: from genomics to mechanism. Oncogene. 2003; 22:7468–85. [PubMed: 14576852]
18. Hoffmeyer S, Burk O, von Richter O, Arnold HP, Brockmoller J, Johne A, et al. Functional polymorphisms of the human multidrug-resistance gene: multiple sequence variations and correlation of one allele with P-glycoprotein expression and activity in vivo. Proc Natl Acad Sci U S A. 2000; 97:3473–8. [PubMed: 10716719]

Author Manuscript Author Manuscript Author Manuscript Author Manuscript

19. Kim RB, Leake BF, Choo EF, Dresser GK, Kubba SV, Schwarz UI, et al. Identification of functionally variant MDR1 alleles among European Americans and African Americans. Clin Pharmacol Ther. 2001; 70:189–99. [PubMed: 11503014]

20. Tang K, Ngoi SM, Gwee PC, Chua JM, Lee EJ, Chong SS, et al. Distinct haplotype profiles and strong linkage disequilibrium at the MDR1 multidrug transporter gene locus in three ethnic Asian populations. Pharmacogenetics. 2002; 12:437–50. [PubMed: 12172212]

21. Marzolini C, Paus E, Buclin T, Kim RB. Polymorphisms in human MDR1 (P-glycoprotein): recent advances and clinical relevance. Clin Pharmacol Ther. 2004; 75:13–33. [PubMed: 14749689]

22. Johne A, Kopke K, Gerloff T, Mai I, Rietbrock S, Meisel C, et al. Modulation of steady-state kinetics of digoxin by haplotypes of the P-glycoprotein MDR1 gene. Clin Pharmacol Ther. 2002; 72:584–94. [PubMed: 12426522]

23. Yi SY, Hong KS, Lim HS, Chung JY, Oh DS, Kim JR, et al. A variant 2677A allele of the MDR1 gene affects fexofenadine disposition. Clin Pharmacol Ther. 2004; 76:418–27. [PubMed: 15536457]

24. Yates CR, Zhang W, Song P, Li S, Gaber AO, Kotb M, et al. The effect of CYP3A5 and MDR1 polymorphic expression on cyclosporine oral disposition in renal transplant patients. J Clin Pharmacol. 2003; 43:555–64. [PubMed: 12817518]

25. Kimchi-Sarfaty C, Oh JM, Kim IW, Sauna ZE, Calcagno AM, Ambudkar SV, et al. A “silent” polymorphism in the MDR1 gene changes substrate specificity. Science. 2007; 315:525–8. [PubMed: 17185560]

26. Brimacombe KR, Hall MD, Auld DS, Inglese J, Austin CP, Gottesman MM, et al. A dual-fluorescence high-throughput cell line system for probing multidrug resistance. Assay Drug Dev Technol. 2009; 7:233–49. [PubMed: 19548831]

27. Sauna ZE, Muller M, Peng XH, Ambudkar SV. Importance of the conserved Walker B glutamate residues, 556 and 1201, for the completion of the catalytic cycle of ATP hydrolysis by human P-glycoprotein (ABCB1). Biochemistry. 2002; 41:13989–4000. [PubMed: 12437356]

28. Ambudkar SV. Drug-stimulatable ATPase activity in crude membranes of human MDR1-transfected mammalian cells. Methods Enzymol. 1998; 292:504–14. [PubMed: 9711578]

29. Ohnuma S, Chufan E, Nandigama K, Jenkins LM, Durell SR, Appella E, et al. Inhibition of multidrug resistance-linked P-glycoprotein (ABCB1) function by 5′-fluorosulfonylbenzoyl 5′-adenosine: evidence for an ATP analogue that interacts with both drug-substrate-and nucleotide-binding sites. Biochemistry. 2011; 50:3724–35. [PubMed: 21452853]

30. Hull RN, Cherry WR, Weaver GW. The origin and characteristics of a pig kidney cell strain, LLC-PK. In Vitro. 1976; 12:670–7. [PubMed: 828141]

31. Ito S, Woodland C, Harper PA, Koren G. P-glycoprotein-mediated renal tubular secretion of digoxin: the toxicological significance of the urine-blood barrier model. Life Sci. 1993; 53:PL25–31. [PubMed: 8100040]

32. Cornwell MM, Pastan I, Gottesman MM. Certain calcium channel blockers bind specifically to multidrug-resistant human KB carcinoma membrane vesicles and inhibit drug binding to P-glycoprotein. J Biol Chem. 1987; 262:2166–70. [PubMed: 2434476]

33. Kanamaru H, Kakehi Y, Yoshida O, Nakanishi S, Pastan I, Gottesman MM. MDR1 RNA levels in human renal cell carcinomas: correlation with grade and prediction of reversal of doxorubicin resistance by quinidine in tumor explants. J Natl Cancer Inst. 1989; 81:844–9. [PubMed: 2724349]

34. Fung KL, Gottesman MM. A synonymous polymorphism in a common MDR1 (ABCB1) haplotype shapes protein function. Biochim Biophys Acta. 2009; 1794:860–71. [PubMed: 19285158]

35. Plotkin JB, Kudla G. Synonymous but not the same: the causes and consequences of codon bias. Nat Rev Genet. 2010; 12:32–42. [PubMed: 21102527]

36. Sauna ZE, Kimchi-Sarfaty C. Understanding the contribution of synonymous mutations to human disease. Nat Rev Genet. 2011; 12:683–91. [PubMed: 21878961]

37. Brest P, Lapaquette P, Souidi M, Lebrigand K, Cesaro A, Vouret-Craviari V, et al. A synonymous variant in IRGM alters a binding site for miR-196 and causes deregulation of IRGM-dependent xenophagy in Crohn’s disease. Nat Genet. 2011; 43:242–5. [PubMed: 21278745]

Author Manuscript

Author Manuscript

Author Manuscript

Author Manuscript

38. Bruun GH, Doktor TK, Andresen BS. A synonymous polymorphic variation in ACADM exon 11 affects splicing efficiency and may affect fatty acid oxidation. Mol Genet Metab. 2013; 110:122–8. [PubMed: 23810226]

39. Lawrie DS, Messer PW, Hershberg R, Petrov DA. Strong Purifying Selection at Synonymous Sites in D. melanogaster. PLoS Genet. 2013; 9:e1003527. [PubMed: 23737754]

40. Lai Y, Huang M, Li H, Wang XD, Li JL. Distinct genotype distribution and haplotype profiles in MDR1 gene among Chinese Han, Bai, Wa and Tibetan ethnic groups. Die Pharmazie. 2012; 67:938–41. [PubMed: 23210244]

41. Kimchi-Sarfaty C, Marple AH, Shinar S, Kimchi AM, Scavo D, Roma MI, et al. Ethnicity-related polymorphisms and haplotypes in the human ABCB1 gene. Pharmacogenomics. 2007; 8:29–39. [PubMed: 17187507]

42. Mechetner EB, Roninson IB. Efficient inhibition of P-glycoprotein-mediated multidrug resistance with a monoclonal antibody. Proc Natl Acad Sci U S A. 1992; 89:5824–8. [PubMed: 1352877]

43. Kim H, Barroso M, Samanta R, Greenberger L, Sztul E. Experimentally induced changes in the endocytic traffic of P-glycoprotein alter drug resistance of cancer cells. Am J Physiol. 1997; 273:C687–702. [PubMed: 9277367]

44. Siegsmund M, Brinkmann U, Schaffeler E, Weirich G, Schwab M, Eichelbaum M, et al. Association of the P-glycoprotein transporter MDR1(C3435T) polymorphism with the susceptibility to renal epithelial tumors. J Am Soc Nephrol. 2002; 13:1847–54. [PubMed: 12089380]

45. Hitzl M, Drescher S, van der Kuip H, Schaffeler E, Fischer J, Schwab M, et al. The C3435T mutation in the human MDR1 gene is associated with altered efflux of the P-glycoprotein substrate rhodamine 123 from CD56+ natural killer cells. Pharmacogenetics. 2001; 11:293–8. [PubMed: 11434506]

46. Dalton WS, Durie BG, Alberts DS, Gerlach JH, Cress AE. Characterization of a new drug-resistant human myeloma cell line that expresses P-glycoprotein. Cancer Res. 1986; 46:5125–30. [PubMed: 2875788]

47. Ambudkar SV, Kim IW, Xia D, Sauna ZE. The A-loop, a novel conserved aromatic acid subdomain upstream of the Walker A motif in ABC transporters, is critical for ATP binding. FEBS Lett. 2006; 580:1049–55. [PubMed: 16412422]

48. Tsai CJ, Sauna ZE, Kimchi-Sarfaty C, Ambudkar SV, Gottesman MM, Nussinov R. Synonymous Mutations and Ribosome Stalling Can Lead to Altered Folding Pathways and Distinct Minima. J Mol Biol. 2008; 383:281–91. [PubMed: 18722384]

Author Manuscript
Author Manuscript
Author Manuscript
Author Manuscript

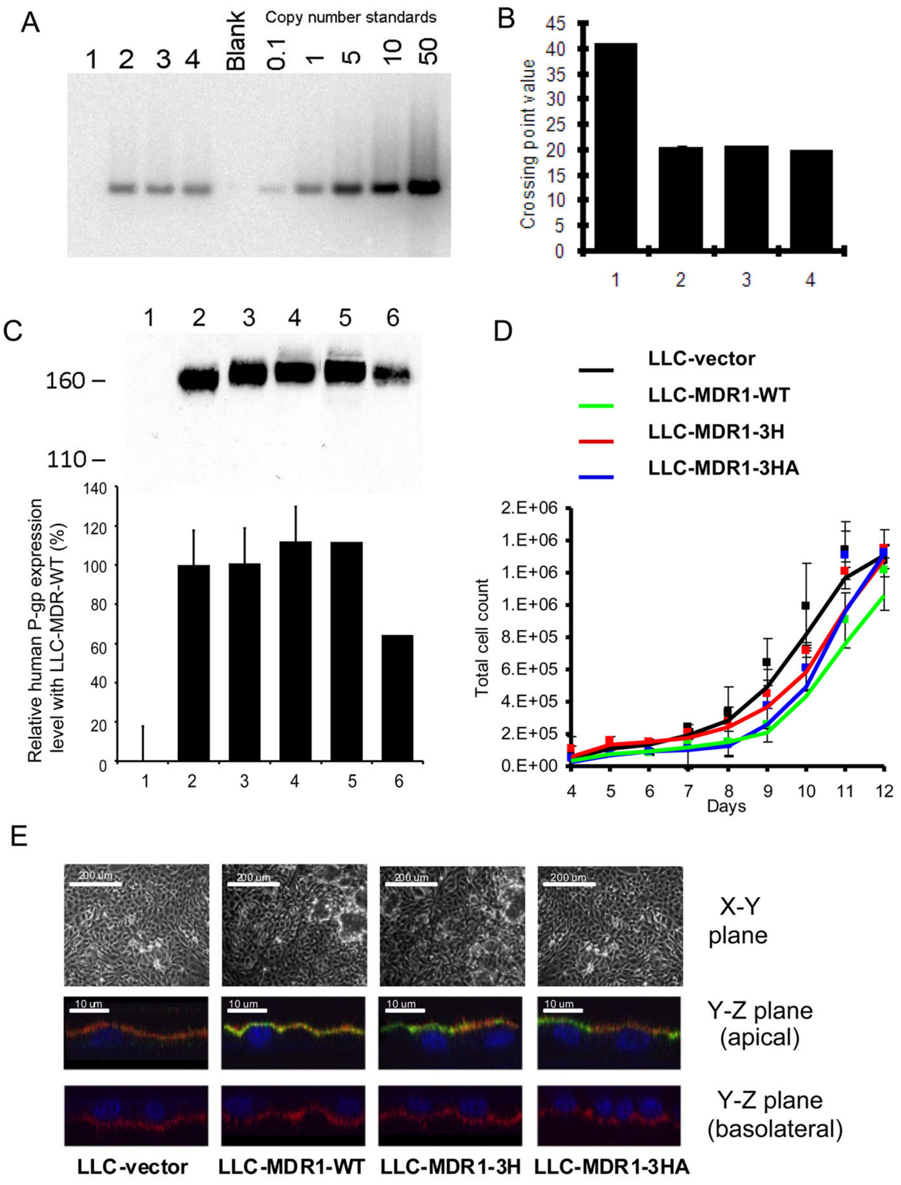

Figure 1.

Characterization of LLC-MDR1 cell lines. A, Copy number determination by Southern blot analysis. Genomic DNA samples were run with a blank control and copy number standards. B, qRT-PCR analysis. The mean values are the average of 3 experiments and are plotted in the histogram. C, Recombinant P-gp expression by Western analysis. LLC-vector (lane1), LLC-MDR1-WT (lane 2), LLC-MDR1-3H (lane 3), LLC-MDR1-3HA (lane 4), KB-V1 (lane 5), and KB-8-5 (lane 6) are shown. The bar chart shows relative expression level of P-gp in each cell line calculated by densitometry analysis. D, Cellular morphology and localization of P-gp. Top: Cells visualized by light microscopy. Bottom: X–Z plane of the LLC-MDR1 cells. Cell monolayers were labeled with MRK16 (green, for P-gp), biotin-strepavdin-PE (red, for plasma membrane) and DAPI (blue, for nucleus). E, LLC-MDR1 cell lines have comparable growth rates. LLC-vector (black), LLC-MDR1-WT (green),

Author Manuscript
Author Manuscript
Author Manuscript
Author Manuscript

LLC-MDR1-3H (red), LLC-MDR1-3HA (blue) cells are shown. Each point in the chart represents an average cell number from 3 samples.

Author Manuscript

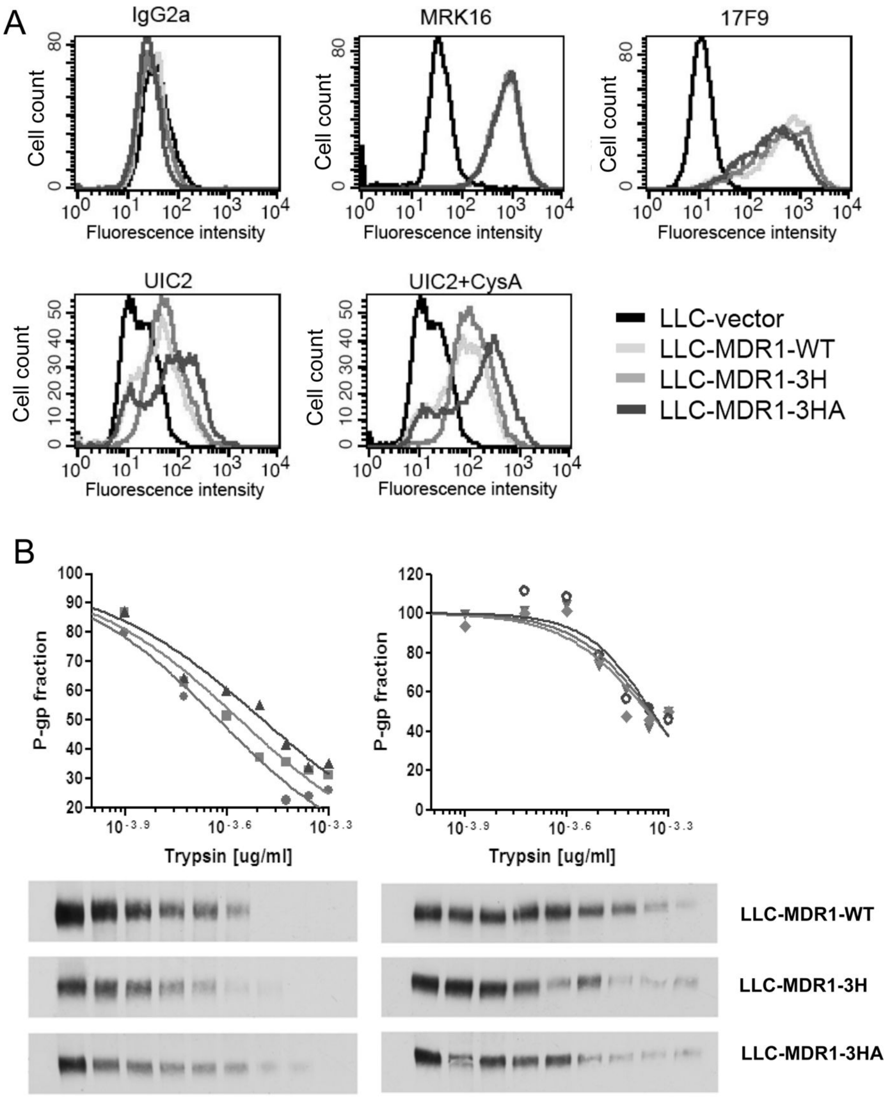

Figure 2.

Stably-expressed haplotype and mutant P-gps have altered protein conformations. A, FACS analysis of cells labeled with different anti-Pgp antibodies. Each histogram represents at least 2 independent experiments. B, Sensitivity of MDR1 wild-type (circle), haplotype (square), and mutant P-gp (triangle) to trypsin. Crude membranes prepared from the cell lines were treated with, from left to right, 0.000, 0.625, 1.250, 1.875, 2.500, 3.125, 3.750, 4.375, and 5.000 trypsin (μg). Charts and Western blots with trypsin (left) and with trypsin + 20 μM verapamil (right) are shown. The upper panel shows quantified data from Western blots shown below. Each point in the plot represents an average cell number from 3 independent experiments.

Author Manuscript
Author Manuscript
Author Manuscript

Author Manuscript Author Manuscript Author Manuscript Author Manuscript

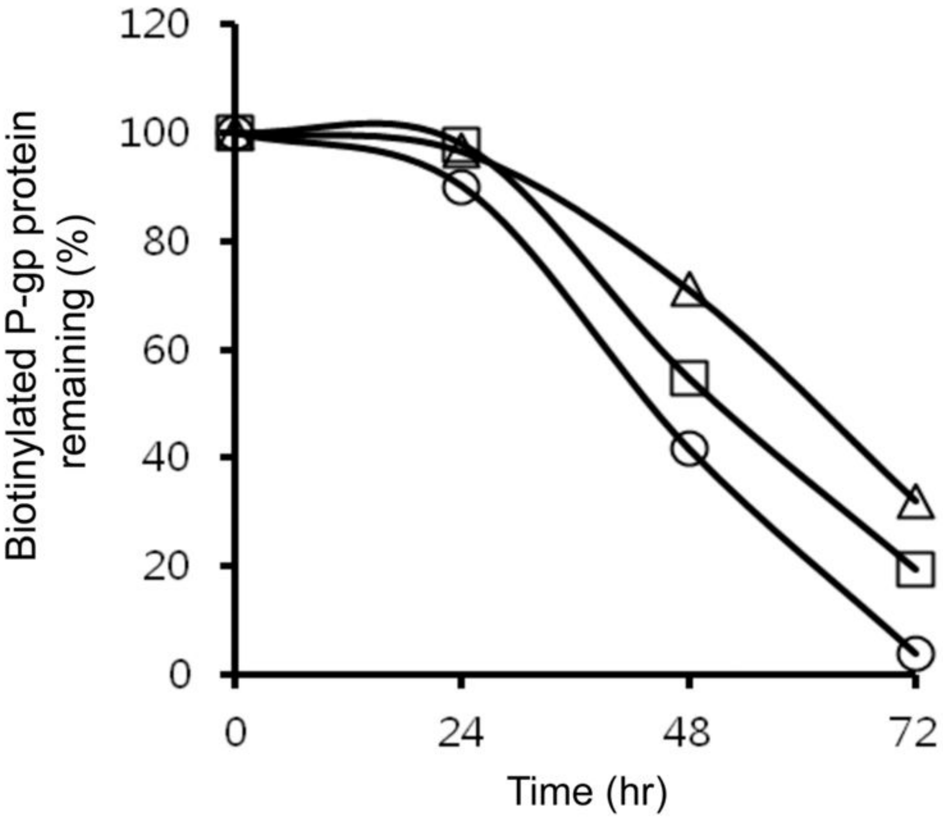

Figure 3.

Mutant and haplotype P-gps remain longer on the cell surface. LLC-MDR1-WT (circles), LLC-MDR1-3H (squares) and LLC-MDR1-3HA (triangles) cells were labeled with biotin and MRK16 for FACS analysis. The percentage of biotin-labeled MRK16 positive cells remaining at each time point was plotted. Each point in the plot represents an average cell number from 3 independent experiments.

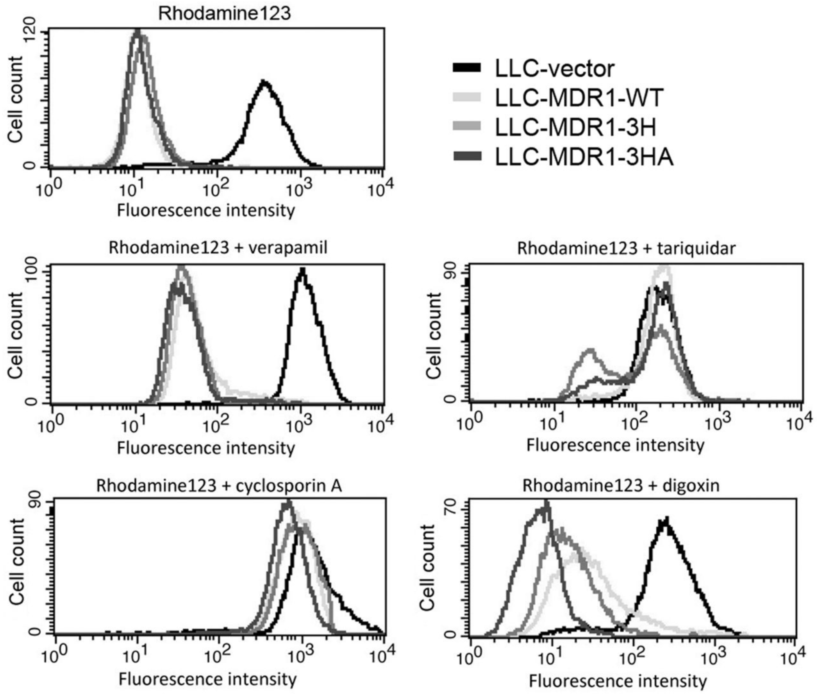

Figure 4.
Variant P-gps exhibit different drug efflux functions in a substrate-dependent manner. Cells were incubated with rhodamine 123 for 20 min at 37°C, followed by 40 min efflux at 37°C and then FACS analysis. Assays were repeated at least 3 times.

Author Manuscript
Author Manuscript
Author Manuscript
Author Manuscript

Author Manuscript Author Manuscript Author Manuscript Author Manuscript

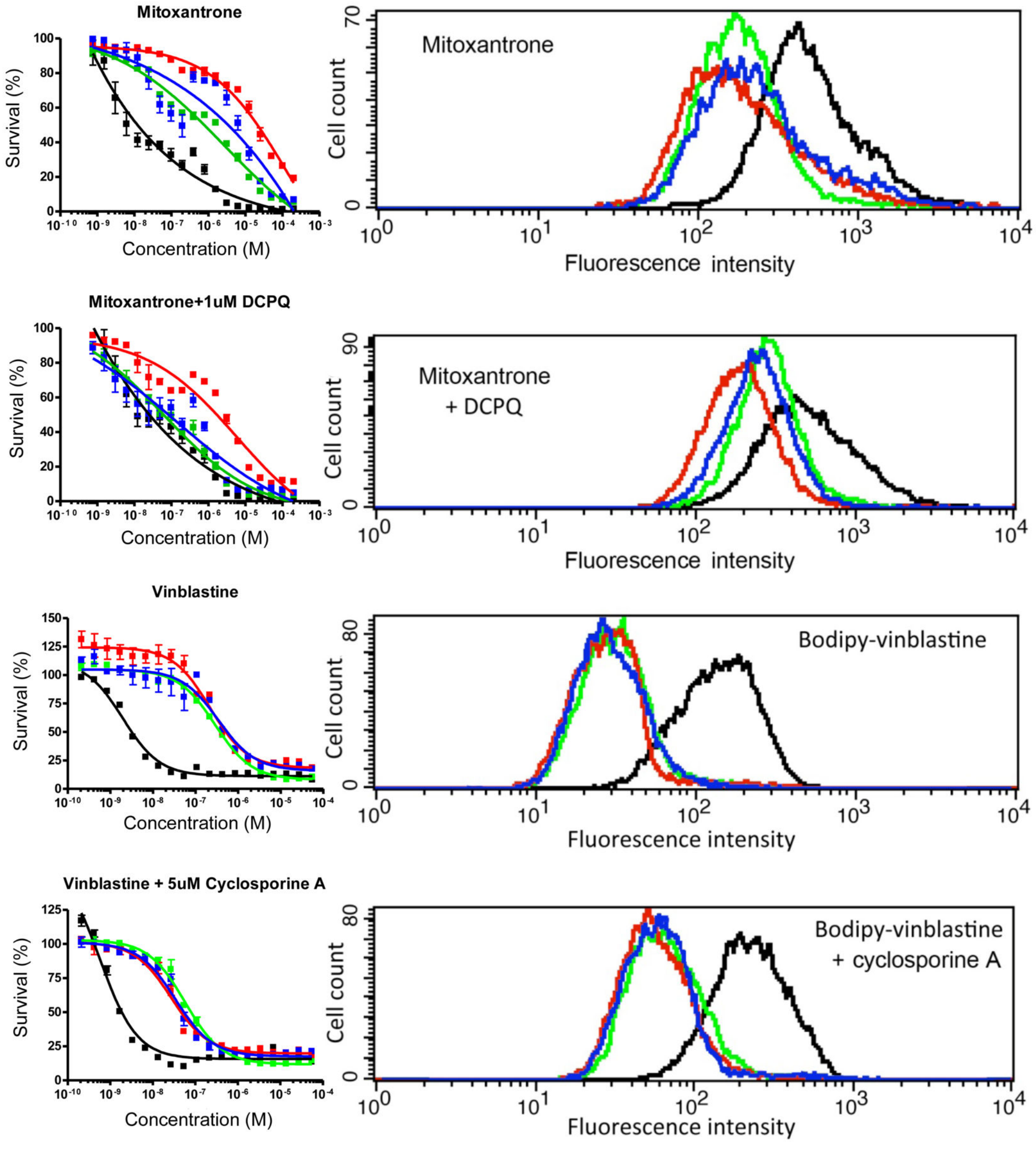

Figure 5.

P-gp transfected cell lines show differential sensitivity to cytotoxic drugs. LLC-vector (black), LLC-MDR1-WT (green), LLC-MDR1-3H (red), LLC-MDR1-3HA (blue) cells were tested with cytotoxicity assays (left panel) and drug efflux assays (right panel). For cytotoxicity assays, cells were incubated with increasing concentrations of drugs for 72 hrs. For drug accumulation-efflux assays, cells were incubated with mitoxantrone ± DCPQ for 30 min followed by 45 min efflux. For bodipy-vinblastine ± cyclosporine A, cells were incubated for 20 min followed by 30 min efflux. Assays were repeated at least 3 times. Each point in the cytotoxicity assay charts represents an average cell number from 3 independent experiments.

Author Manuscript

Author Manuscript

Author Manuscript

Author Manuscript

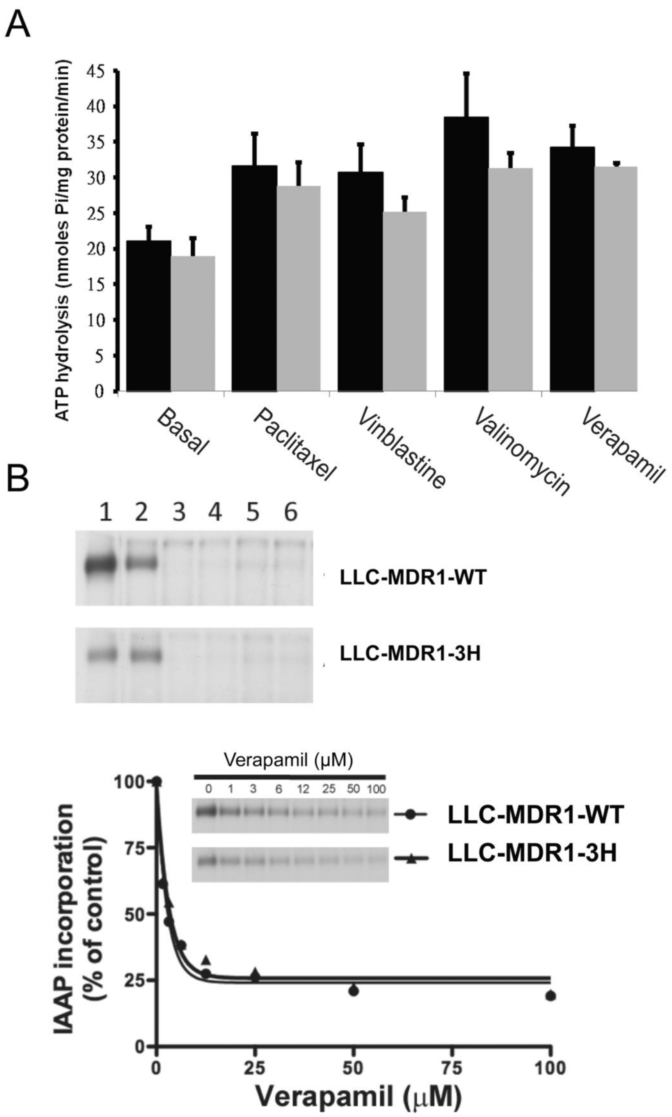

Figure 6.

P-gp variant forms do not differ from wild-type in ATPase and photoaffinity labeling Assays. A, Substrate-stimulated ATPase activities using crude membranes from LLC-MDR1-WT (black) and LLC-MDR1-3H (gray) cells. B, [^{125}I]-IAAP Photoaffinity labeling of P-gp wild-type and haplotype in the absence (lane 1) and the presence of 10 μM tasigna (lane 2), 10 μM verapamil (lane 3), 10 μM tariquidar (lane 4), 10 μM vinblastine (lane 5), and 5 μM paclitaxel (lane 6). The scatter plot shows the incorporation of [^{125}I]-IAAP in LLC-MDR1-WT (circle) and LLC-MDR1-3H (triangle) membrane proteins with verapamil from 0 to 100 μM. Autoradiographs from representative experiments are shown. Similar results were obtained in two additional experiments.

Author Manuscript Author Manuscript Author Manuscript Author Manuscript

Table 1

Cytotoxicity of selected substrates and inhibitors of P-gp in wild-type and haplotype-expressing LLCPK1 stable cell lines

Drug	Inhibitor	IC_{50} (μM)[a]				Resistance ratio (fold)[b]			
		LLC-vector	LLC-MDR1-WT	LLC-MDR1-3H	LLC-MDR1-3HA	LLC-vector	LLC-MDR1-WT	LLC-MDR1-3H	LLC-MDR1-3HA
**Mitoxantrone**	-	0.01 ± 0.003	1.5 ± 0.3	14.6 ± 1.9	9.2 ± 2.5	1	114	1138	718
**Mitoxantrone**	**DCPQ**	0.065 ± 0.015	0.2 ± 0.04	3.2 ± 0.6	0.8 ± 0.2	1	4	50	12
**Vinblastine**	-	0.002 ± 0.0002	0.3 ± 0.02	0.2 ± 0.03	0.3 ± 0.06	1	146	111	175
**Vinblastine**	**CsA**	0.0006 ± 0.0001	0.06 ± 0.004	0.05 ± 0.003	0.05 ± 0.003	1	101	83	95

[a] The IC_{50} values were calculated by non-linear regression analysis in GraphPad Prism, using dose-response data from MTT assay.

[b] The resistance ratio is the fold of IC_{50} values relative to the LLC-vector cells.
