# Supplementary material for: Computational Analysis of MDR1 Variants Predicts Effect on Cancer Cells via their Effect on mRNA Folding
Source: PLoS Comput Biol. 2024 Dec 26;20(12):e1012685. doi: 10.1371/journal.pcbi.1012685 (PMC11670953; doi:10.1371/journal.pcbi.1012685)
Supplement: Attachment — Submitted filename: Nusssinov. JMB. protein folding.pdf [file pcbi.1012685.s018.pdf]

doi:10.1016/j.jmb.2008.08.012 J. Mol. Biol. (2008) **383**, 281–291

JMB

Available online at www.sciencedirect.com

ScienceDirect

ELSEVIER

REVIEW

# Synonymous Mutations and Ribosome Stalling Can Lead to Altered Folding Pathways and Distinct Minima

**Chung-Jung Tsai[1], Zuben E. Sauna[2], Chava Kimchi-Sarfaty[2], Suresh V. Ambudkar[2], Michael M. Gottesman[2] and Ruth Nussinov[1,3]***

[1]*Basic Research Program, SAIC–Frederick, Inc., Center for Cancer Research Nanobiology Program, NCI–Frederick, Frederick, MD 21702, USA*

[2]*Laboratory of Cell Biology, Center for Cancer Research, National Cancer Institute, Bethesda, MD 20892, USA*

[3]*Department of Human Genetics and Molecular Medicine, Sackler Institute of Molecular Medicine, Sackler School of Medicine, Tel Aviv University, Tel Aviv 69978, Israel*

*Received 2 April 2008; received in revised form 31 July 2008; accepted 6 August 2008*
Available online 12 August 2008

***Edited by M. Levitt***

How can we understand a case in which a given amino acid sequence folds into structurally and functionally distinct molecules? Synonymous single-nucleotide polymorphisms in the *MDR1* (*multidrug resistance 1* or *ABCB1*) gene involving frequent-to-rare codon substitutions lead to identical protein sequences. Remarkably, these alternative sequences give a protein product with similar but different structures and functions. Here, we propose that long-enough ribosomal pause time scales may lead to alternate folding pathways and distinct minima on the folding free energy surface. While the conformational and functional differences between the native and alternate states may be minor, the *MDR1* case illustrates that the barriers may nevertheless constitute sufficiently high hurdles in physiological time scales, leading to kinetically trapped states with altered structures and functions. Different folding pathways leading to *conformationally similar* trapped states may be due to swapping of (fairly symmetric) segments. Domain swapping is more likely in the no-pause case in which the chain elongates and folds simultaneously; on the other hand, sufficiently long pause times between such segments may be expected to lessen the chances of swapping events. Here, we review the literature in this light.
© 2008 Elsevier Ltd. All rights reserved.

*Keywords:* synonymous mutations; protein folding; kinetics; multidrug resistance gene; ABC transporter

Recently, Kimchi-Sarfaty *et al.* observed that synonymous single-nucleotide polymorphisms (SNPs) that retain the amino acid sequence can nevertheless result in a protein with an altered structure and function.[1] Synonymous SNPs are silent mutations that, if occurring in protein-coding regions, involve nucleotide substitutions still coding for the same amino acid. Since the genetic code is degenerate, many types of such SNPs can be found. Hence, when examined from the standpoint of the stationary amino acid sequence, no change is observed. Yet, in the case of the *MDR1* (*multidrug resistance 1*) gene, the article reports that despite the identical protein sequence, certain combinations of synonymous SNPs involving frequent-to-rare codon substitutions appear to alter the transport substrate binding site shape and substrate specificity of the gene product P-glycoprotein (P-gp). Rare codons may result in ribosome stalling,[1,2] due to either a lower concentration of cognate tRNAs or an alteration of the RNA structure. Translational stalling enforces sequential folding pathways that may lead to different distinct minima in the folding free energy landscape. The

**Corresponding author.* Basic Research Program, SAIC–Frederick, Inc., Center for Cancer Research Nanobiology Program, NCI–Frederick, Frederick, MD 21702, USA. E-mail address: ruthn@ncifcrf.gov.

Abbreviations used: SNP, single-nucleotide polymorphism; *MDR1*, *multidrug resistance 1*; P-gp, P-glycoprotein; ABC, ATP-binding cassette; OA, ovalbumin; OAR, renatured OA; OAN, native OA; CFTR, cystic fibrosis transmembrane conductance regulator; NBD, nucleotide binding domain.

0022-2836/$ - see front matter © 2008 Elsevier Ltd. All rights reserved.

observations that some inhibitors bind to one of P-gp conformational states but not another, that single-residue substitutions may dramatically change substrate specificity,[2] and that the P-gp recognizes hundreds of substrates and inhibitors in a large pleomorphic substrate–inhibitor interaction site suggest that the conformational change between the P-gp states is limited. It is unclear as to which of the two states is the "native" and which is the "mutant": the haplotype (1236C>T/2677T>A/3435C>T) described in the study by Kimchi-Sarfaty *et al.*[1] is fully functional as a drug transporter, approaching 50% in certain ethnic populations, thus suggesting some substrate-selective advantage. The SNPs that constitute the haplotype have been the subject of many studies, and 3435C>T (at exon 26) is the most commonly implicated in the diverse responses of patients toward a long list of P-gp substrates. These polymorphisms are risk factors for several diseases[3–6] and may affect the progression of others.[7–10] In addition, the pharmacokinetics of several drugs, such as cyclosporine A,[11] nelfinavir,[12] fexofenadine,[13] and digoxin,[14] are affected by these SNPs. This is not surprising because numerous mutational studies of P-gp have demonstrated that point mutations in mammalian P-gp's affect substrate specificity (see Table 1 in the work of Ambudkar *et al.*[15] for an extensive list).

## The case of the *MDR1* gene

The *MDR1* gene product P-gp, an ATP-binding cassette (ABC) transporter, is an ATP-driven efflux pump for many substrate hydrophobic drugs that enter the cell via diffusion through the membrane.[2] Consequently, it is hypothesized to play a major role in multidrug resistance during chemotherapy in metastatic cancers. Haplotype polymorphisms coded by double or triple combinations of mutations (C1236T–G2677T, C1236T–C3435T, G2677T–C3435T, and C1236T–G2677T–C3435T) were less effective against cyclosporine A and verapamil inhibitors, while no change was observed in the transport of a range of substrates for the single polymorphisms of C1236T, C3435T, and G2677T.[1] Thus, it is not the nonsynonymous polymorphism at G2677T that is responsible for the altered functional inhibition of P-gp: the combination of C3435T, a synonymous polymorphism, with C1236T, C2677T, or the C1236T/C2677T haplotype results in altered specificity. The altered transport implicated a structural change. The binding to conformation-sensitive monoclonal antibodies (UIC2) and trypsin digestion patterns supported such a conclusion. It is also unlikely that the observed altered specificity could be explained by differences in glycosylation of the wild type and haplotype. P-gp's that were expressed in mammalian cells and immunoblots show that the wild type and haplotype have identical molecular masses. Furthermore, previous studies have demonstrated that glycosylation does not affect the function of P-gp.[16] Thus, the altered haplotype function is not likely to be due to altered glycosylation. Furthermore, (i) cyclosporine and verapamil inhibition differences between the wild type and the C1236T–G2677T–C3435T haplotype increased with the mutant DNA concentration, suggesting a relationship with mRNA levels; (ii) there was no rapamycin inhibition difference between the two species; and (iii) the SNP, although silent, involved frequent-to-rare codon substitutions.

There is substantial evidence that P-gp exists and functions as a monomer.[17] Currently, no structure exists for the P-gp transporter. However, structures of bacterial MDR proteins and their cognate regulators provide a wealth of molecular detail about how these proteins recognize multiple substrates. For example, structures of QacR, the multiple-drug binding protein that represses the transcription of QacA, have been solved individually, with six substrates and with two drugs bound simultaneously.[18–20] These results are broadly consistent with the structure of the *Escherichia coli* AcrB pump solved with four substrates.[21] All these structures show the use of a single large cavity comprising a "drug-binding pocket" in which individual "drug-binding sites" are generated by subtle alterations in the accessibility of different subsets of residues for drug binding. What these studies clearly demonstrate are that MDR proteins can alter substrate specificity fairly easily and that even subtle changes in the conformation of the drug-binding pocket can result in changes in substrate specificity. Moreover, such changes can be measured using several well-established assays.[22]

## Two types of protein folding scenarios to obtain alternate conformations

In both P-gp and amyloids,[23–25] under physiological conditions, some fraction of the molecules is in an alternate conformation. However, the similarity ends there: In the amyloid case, via conformational selection,[24] monomers in this conformation may assemble to create the typical cross-β-structure.[26] If the assembly is below a certain seed size (at least four monomers), the molecules will dynamically associate and disassociate; as the seed grows, stability increases. This kinetic behavior is characteristic of polymerization reactions, suggesting analogy with a nucleated polymerization mechanism.[27,28] Seed formation constitutes the aggregation bottleneck. Fibril extension occurs on a much faster time scale than seed formation. Binding and amyloid growth will shift the equilibrium toward this misfolded conformational state.[24] In contrast, in the second P-gp scenario, the altered state relates to protein synthesis and ribosome pausing effects. Depending on the pause time scales, pause site location, and the protein sequence, such events can lead to alternate folding pathways.

Consequently, there are two types of scenarios: the first involves a transition from the folded state to the misfolded and aggregated state;[29] the second relates to a protein folding pathway. The co-translational folding pathway with altered kinetics and intermediate states[30–32] can lead to functionally distinct

proteins. For both cases, the altered conformations need not involve the entire molecules. For amyloids, a local segment may adopt an extended state, with the rest of the structure retaining a native-like state. For the ribosome stalling case, the downstream chain might fold into its native conformation. Whether such kinetically controlled scenarios via rare codon usage are used in nature to regulate function is unclear. As we argue below, the chances of evolution adopting a kinetic control as a regulatory mechanism to obtain *globally* different conformations for a given sequence are fairly low due to the lack of robustness and the risk involved. On the other hand, a *limited* change, with the conformations lying nearby on a rugged folding landscape funnel bottom, is likely, allowing them to bind to a broader range of ligands.

Thus, the folded-to-misfolded transition and aggregation that are observed in neurodegenerative diseases are reflected in a change in the protein folding free energy landscape (Fig. 1a). On the other hand, in ribosome stalling cases, the landscape is unchanged; instead, different folding pathways may lead to distinct minima and alternate (functional or diseased) conformations (Fig. 1b).

## Co-translational (sequential) folding

*In vivo*, evolution via natural selection ensures that the newly synthesized polypeptide chain always folds spontaneously into a native functional conformation with or without help from molecular chaperones. *In vitro*, a small single-domain protein with only one hydrophobic folding unit is always able to fold and unfold reversibly.[33] These two observations suggest that the three-dimensional fold of a protein is determined entirely by its amino acid sequence. In a sequential folding mechanism, contiguous fragments in the one-dimensional chain interact in the three-dimensional fold, significantly reducing the possibility of misfolding.[34–36] On the other hand, in the more "complex" protein folds, substantial contacts between nonsequential pieces of the chain are observed. Sequential folding is advantageous:[36] since it is kinetically more favorable, it is expected to be faster. Chain linkage contributes to bringing mutually stabilizing consecutive building blocks together more frequently than segments that are sequentially far. In terms of the folding funnel free energy landscape theory,[37,38] the folding process should encounter fewer rough bumps on the free energy surface in a sequential folding case. This implies that in a sequential folding mechanism, the likelihood of misfolding is greatly reduced.[36,39]

It is widely believed that with a few exceptions, the native conformations of proteins at physiological temperatures are at their global free energy minima. If this is correct, then misfolding may occur as kinetically trapped alternate conformations, due either to mutations or to changes in folding condi-

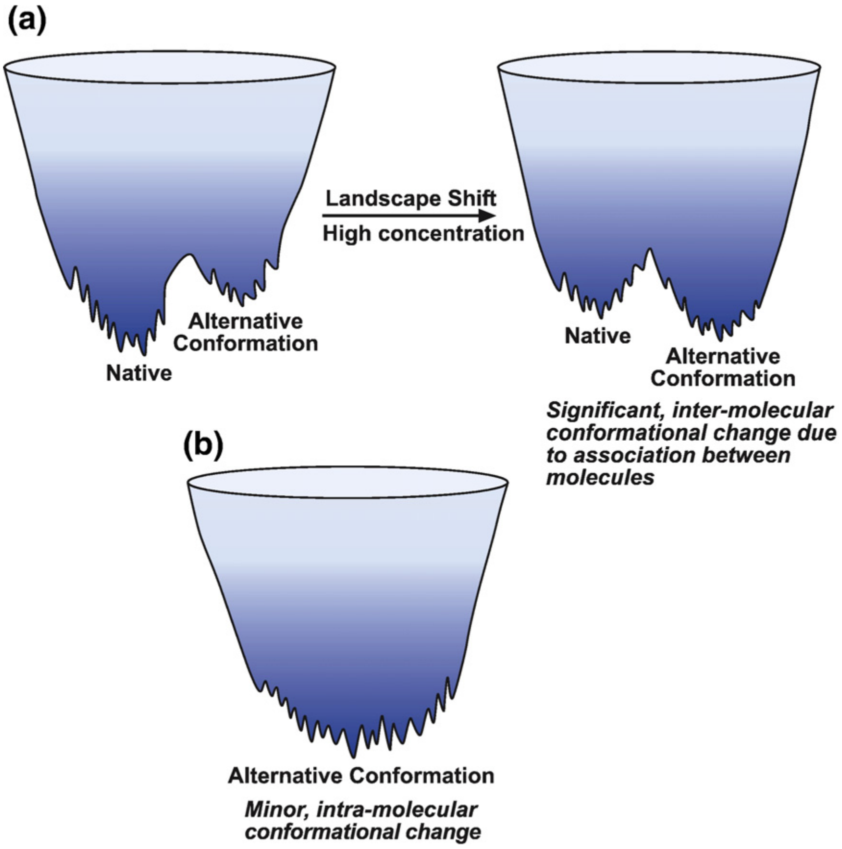


**Fig. 1.** Simplified folding free energy landscape illustrating two types of protein folding pattern scenarios. At the single-molecule level, the native protein conformation is more favorable thermodynamically than the altered conformation due to synonymous mutations (a). However, the folding free energy shifts toward favoring the haplotype conformation at a high concentration with a portion of the conformation changed significantly due to intermolecular association. The sizable barrier in (a) reflects the involvement of a significant conformational change. Panel (b) illustrates the second type of folding pattern. At the bottom of the folding funnel, the landscape is rugged with many local minima, each representing a similar but distinct core structure. The intermediate barrier in (b) emphasizes that there is only a minor conformational change when moving from one local minimum to another. Unlike the first (amyloid) folding pattern, here, the folding free energy landscape will not change since it does not involve intermolecular interactions. The final folded conformation is mainly controlled by folding kinetics: a different folding pathway leads to a different conformation.

tions. Evolution lessened the chances of misfolding by preserving folds that are not only at their thermodynamic global minima but also kinetically favorable. Still, kinetic trapping events may be essential for particular functions, such as in the remarkable serpin protease family[40] case. In protein folding, the strictly sequential interactions between energetically favorable segments fulfill perfectly the requirements for reaching the global minimum without encountering high barriers and ribosome pausing enforces a sequential folding pathway.

## Ribosome pausing effects and folding kinetics

While considerable work focused on protein folding kinetics, uneven translation rates, particularly stalling at given sites over relevant time scales during protein synthesis, have not been thoroughly explored. *In vivo*, folding kinetics relate to two factors: (i) translation rate and evenness as a function of the codon sequence and local mRNA stability (i.e., the mRNA sequence) and (ii) folding rates as determined by protein structural motifs, which under equilibrium conditions are a function of the protein sequence. For the first factor, *in vivo* translation rates may be affected by the ribosome pause time scales; the longer the pause, the more likely an altered kinetic scenario is. Stalling may result from frequent-to-rare synonymous codon substitutions[41–45] due to lower tRNA populations[46] and slower tRNA recharging[42] (the longer the stretches of predominantly rare codons, the longer the pause) or to the local stable mRNA structure[47,48] or codon/anticodon base pairing.[49] Since only a certain fraction of the ribosomes will stall, we expect both native and altered conformational states. For the second factor, a protein is likely to fold slowly if its folding landscape has at least two minima; on the other hand, it is likely to be a fast folder if it has an all-α-fold,[50] undergoes a sequential folding process, and has a low contact order[51] (i.e., where sequentially close residues are in van der Waals contact) that sequential folders usually do. Many of the fast folders are small helical protein subdomains.[50] The folding free energy landscape of fast folders is not expected to have high barriers. Under such circumstances, folding rates relate to energy landscape roughness. β-Structures fold with slower kinetics, since β-sheets have a rougher energy landscape as compared with α-helices; however, β-protein folding rate measurements are scant compared with α-proteins. Ultrafast folders are rarely observed to populate the partially folded state, quickly achieving their native-state conformations.

Let us assume that there is a single or a stretch of silent substitutions at position $S_1$ (Fig. 2) that leads to stalled ribosomes at this site compared with native folding. $S_1$ divides the protein into an upstream part A and a downstream part B, where A and B are protein building blocks.[36,52] If both A and B are fast folders, pausing at $S_1$ is unlikely to lead to altered folding pathways and altered conformations, since both native conformations are obtained rapidly. If both A and B are slow folders, it is also unlikely, since B also needs time to reach its favored state. If A is a fast folder and B is a slow folder, A will fold into its native state regardless of whether the ribosome stalls and the folding of B will be unaffected. On the other hand, if A is a slow folder and B is a fast folder, the situation is different: the slow folder A is likely to have competing conformations, say, $A_1$ and $A_2$. Let us assume that on its own $A_2$ is more stable than the native $A_1$. Under native conditions, the fast folder B may serve as a template to $A_1$ since their association is favorable. On the other hand, if the ribosome stalls, by the time B is synthesized $A_2$ had already formed. While in this case stalling leads to different branches in the folding pathway, all are on the native pathway, leading to minor conformational changes. Here, we related to single-site stalling events. In the *MDR1* case, two or three simultaneous substitutions are observed to lead to such effects, with likely more complex kinetic scenarios.

The statistics relating to the environment and locations of the rare codons is interesting:[39] Studies of *E. coli* observed that (i) fast-folding α-helical sequences tend to have fewer rare codons than the slower-folding β-sheets,[53] which have higher contact order values;[51] (ii) for the *EgFABP1* fatty acid binding protein,[45] removal of the three rare codons in a helix–turn–helix leads to a higher aggregation rate *in vivo* but not *in vitro*; (iii) about 70% of protein domain boundaries in *E. coli* are coded by rare codons;[54] and (iv) there is a correlation between gene length and rare codon frequencies: longer genes tend to have higher fractions of rare codons.[55] Furthermore, while actual kinetic data are scant, the three exceptions point that rare codons have slower translation rates (for example, for the frequent GAA codon, the translation rate is 21.6 codons per second, whereas for the rare GAG codon, the translation rate is 6.4 codons per second;[56]); also, the longer the rare codon stretch, the slower the translation rate.[56–58] Thus, available data uphold the arguments presented above: fast folders avoid rare codons; longer-range secondary structure motifs have a higher chance of containing them to increase "correct" folding probabilities; longer sequences that have higher misfolding chances have higher tendencies to have rare codons; and, significantly, within these, there is a strong tendency for rare codons to be located between domains, leading to pauses and consequently to higher chances of obtaining favored thermodynamic states. Rare codons enforce an order on the folding of the protein. Thus, it follows that ribosome slowing at certain sites may lead to different folding pathways reaching distinct minima with minor conformational changes.

## A role for a template or an "intramolecular chaperone?"

Since native ultrafast folders are generally subdomains of larger proteins, fast-folding domains may assist the folding of large proteins. The recal-

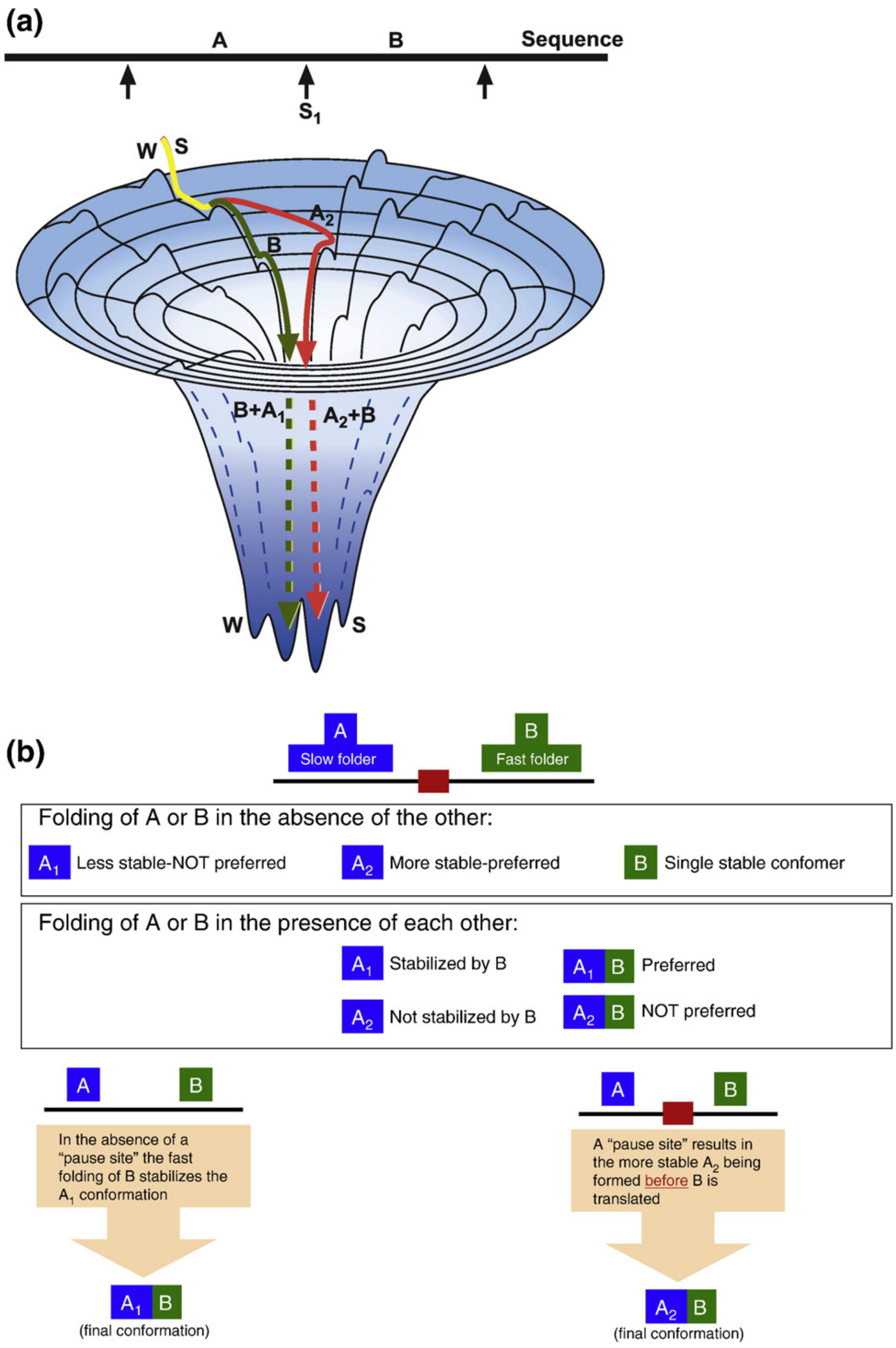


**Fig. 2.** Simple scheme illustrating the origin of a minor conformational change via a ribosome stalling effect. Along a sequence, say, at an arbitrary position $S_1$, fragment A preceding $S_1$ is a slow folder and fragment B following $S_1$ folds faster than fragment A. Also, fragment A has two competing conformations, $A_1$ and $A_2$, with $A_2$ more stable than $A_1$ by itself but $A_1$ becoming more favorable in the presence of fragment B. Let us assume that the folding of the nascent chain is independent. The folding landscape will then be exactly the same for both the wild sequence (W) and the sequence with a synonymous mutation (S) at $S_1$ since they have the same amino acid sequence. The co-translational folding pathway is expected to be identical up to the $S_1$ position. In (a), the folding pathways are drawn as step-by-step arrows on the simplified folding funnel surface. Without a pause at $S_1$, fragment B folds before fragment A; then, fragment A folds on fragment B with an $A_1$ conformation. On the other hand, for the synonymous mutation at the $S_1$ case, the pause enables $A_2$ to fold first, and fragment B follows. The folding branches due to a pause in a sequential folding eventually lead to the bottom of the funnel with minor conformational change between them. Panel (b) provides a diagram of the two folding scenarios.

citrant proteins substantiate such a scenario, in which "assisted folding" takes place via fusion to a poorly folding protein.[59] Fast-folding (sub)domains act as templates or intramolecular chaperones.[52] An intramolecular chaperone catalyzes folding and neither dissociates nor is cleaved. Yet, while intermolecular chaperones catalyze folding by unfolding misfolded conformations or preventing them, an intramolecular chaperone catalyzes folding by binding to, stabilizing, and increasing the populations of native conformations of adjacent fragments. One example of an intramolecular chaperone is the amino-terminal pro-region fragments of some proteases, such as bacterial subtilisin, α-lytic protease, aqualysin, and yeast carboxypeptidase Y, which are cleaved after assisting in the folding.[60] Since pro-regions act as inhibitors covering the enzyme active site, they must be removed for the enzymes to be functional. However, if the fragment is cleaved prior to complete protein synthesis, the chain misfolds. Mixing the cleaved fragment with the remainder of the chain leads to a correctly folded protein.[61,62] An intramolecular chaperone does not work by binding to an intermediate conformation and thereby inducing it to undergo a conformational change to the native state; rather, it binds to the native state via conformational selection[23–25,63] and thereby leads to an equilibrium shift[63] that propagates the binding reaction. In our ribosome pausing scenario case, the fragment labeled as B (Fig. 2) is an intramolecular chaperone, assisting in the folding of A via conformational selection of $A_1$ rather than unfolding $A_2$. Two-domain spectrin[64] presents a similar cooperative scenario. While mechanistically intramolecular chaperones and pro-region segments are similar, the (inhibitory) pro-region function leads to a difference: proteins folding with pro-region assistance are under kinetic control; on the other hand, proteins with uncleaved intramolecular chaperone are under thermodynamic control.

## Ribosome stalling and co-translational folding: some examples

Numerous examples illustrate that co-translational folding reduces the chance of misfolding and that faster protein synthesis rates increase the chance of "incorrectly" folded proteins. When the protein synthesis rate is fast, the entire chain is already synthesized when the protein folds. An early example relates to ovalbumin (OA).[65] Renatured OA (OAR) was separated from the native form (OAN), suggesting a "preferred" pathway leading to the native state. Going back to our scenarios (Figs. 1b and 2), since in renaturation the entire sequence is already synthesized, the refolding of the OAR state resembles the case with no ribosomal pause at $S_1$ *versus* the likely sequential folding of OAN. Replacement of 16 consecutive rare codons by frequent codons in *E. coli CAT* (*chloramphenicol acetyltransferase*) gene leads to protein synthesis rate acceleration and 20% specific activity reduction.[44] Hence, as in OAR, conformations are caught in energy minima in the rugged funnel bottom. In globin,[66] a ribosome-bound nascent 86-residue fragment already attains its native favored state, implicating co-translational folding. Silent mutation effects have also been observed: the *TRP3* gene encodes a bifunctional protein, with anthranilate synthase II and indoglycerol-phosphate synthase activities.[67] Replacement of 10 consecutive rare codons by frequent codons in the second region led to reduced activity,[43,67] suggesting conformational change. Synonymous rare-to-frequent codon substitutions were introduced into the *EgFABP1* (*Echinococcus granulosus fatty acid binding protein*) gene,[45] leading to *in vivo* aggregation. Concentration is critical: in a low concentration, even fast synthesis is likely to yield proteins in their favored states; in contrast, if the concentration is high, the chances of aggregation are higher.

While there is no direct information relating to P-gp folding, another ABC transporter, the cystic fibrosis transmembrane conductance regulator (CFTR), was shown to fold co-translationally.[68] With the C-terminus truncated, the domains still form well-defined structures. CFTR folds during translation, translocation, and membrane insertion, implying slow co-translational folding. Deletion of Phe508 in the nucleotide binding domain (NBD) 1 causes limited alteration in NBD1;[69] however, it disrupts the folding of NBD2. At the earliest biosynthetic stages, wild-type and ΔPhe508 CFTR are susceptible to degradation (all of ΔPhe508 CFTR and 45%–80% of wild-type CFTR), possibly indicating that they still have not attained their native conformation.[70,71] The results of Varga *et al.*[72] suggest a small conformational change.

## The "native" and "altered" conformational states

Above, we focused on a case in which a slowed translation could lead to a protein product with an *altered conformation*, whereas the examples we provided address cases in which accelerated translation leads to aggregation or *reduced activity*. Are these two scenarios different sides of the same coin?

When studying an enzyme, the distinction between "correctly folded" and "misfolded" is relatively straightforward: as a general rule, the binding pocket is specific for a given substrate. On the other hand, in the case of promiscuous transporters, such as those belonging to the ABC family, this distinction is difficult to make. The most detailed structural view comes from the few structures of regulatory proteins of bacterial multiple-drug transporters. These studies show a large drug-binding pocket that can accommodate several substrates, and different drugs use a different subset of residues. The interactions can be quite complex and often counter-intuitive. To give an example, in QacR, the repressor of *qacA*, although different drugs use a different subset of residues, most drugs contact one or more of the four glutamate residues.[19,73,74] The events that follow initiation of translation, protein translocation,

folding, and degradation, are facilitated by molecular chaperones. Soluble proteins are translocated into the endoplasmic reticulum lumen, whereas transmembrane proteins are co-translationally integrated into the lipid bilayer.[75] The degradation of misfolded proteins is as important as the translocation and folding of proteins, and in membrane proteins, this involves an additional level of complexity—that is, the retrotranslocation of proteins from the endoplasmic reticulum into the cytosol.[76] If it is not degraded, it can lead to disease. Thus, the misfolding of a mutant form of the ABC transporter, CFTR, is responsible for cystic fibrosis. The chaperones and cofactors involved in the folding and degradation of this protein have been studied in considerable detail.[77] Given the complexity of this system, it can be argued that in the case of the haplotype, a misfolded protein escapes the quality control machinery. We, on the other hand, would suggest that the haplotype generates a protein that is not misfolded but an alternative conformation. Again, in this situation (relatively unique to P-gp), it is difficult to define the native conformation and thus what is misfolding. It is probably precisely for this reason that the so-called misfolded protein escapes the quality control machinery. We emphasize that the levels of wild-type protein and haplotype at the cell surface are comparable;[1] if the protein synthesized from the haplotype were misfolded and "escaped" the quality control machinery of the cell, only a fraction of the misfolded protein would be expressed at the cell surface. That is, if it were misfolded, it would be translocated and degraded, leading to a lower concentration.

## The origin of minor conformational changes: domain swapping?

It is generally accepted that the protein folding free energy landscape has a funnel-like shape. Visualizing the protein folding process as a combinatorial assembly of building blocks[78,79] can describe all major folding pathways. The order in which the building blocks fold toward the final native conformation of the protein constitutes a particular folding pathway. If the surface at the bottom of the funnel is smooth, there is only one native conformation and all folding pathways lead to it; if the funnel bottom is rugged, different pathways may lead to distinct structures separated by barriers that are not so easy to surmount (Fig. 2). This is the origin of minor conformational changes for (i) sequential *in vivo* folding *versus* folding↔unfolding experiments *in vitro* and (ii) a ribosome stalling mechanism. Under such circumstances, preferred folding pathways are altered, leading to minor conformational changes.

In co-translational protein folding, the energy landscapes vary. Co-translational folding scenarios consider events as the chain elongates, thus increasing the formal number of conformational degrees of freedom. It is intriguing to consider which type of phenomena would retain similar functional sites and functional mechanisms as observed in the P-gp while at the same time be due to altered pathways—that is, which phenomena would lead to two similar conformations that are separated by physiologically sufficiently high barriers that will not be easily crossed. One such phenomenon is *intramolecular domain swapping*. In a domain-swapping mechanism in homodimers, one segment in one monomer is replaced by an equivalent segment from the identical chain in the dimer.[80,81] For some proteins, such as the bovine seminal ribonuclease, it was proposed that both conformations coexist: one with a swapped N-terminal segment and the other without segment swapping.[82,83] It was further suggested that the swapping may have arisen at a later stage in evolution due to functional needs.

In our case, it would be reasonable to consider a scenario in which segment swapping occurred in the faster folder native conformer, unlike in the slower mutant folder. A segment-swapping event would lead to similar conformations, with the only difference being a swapped segment; however, there would be difficult-to-cross barriers in biological time scales to alter the locations of these segments. In the bacterial ABC transporter homodimer,[84] it is reasonable to assume that under appropriate conditions, such as a high concentration, swapping of the last 44 residues at the C-terminal segment may occur between the two monomers (residues 535–578; with the structure consisting of two α-helices, a β-turn, and a third α-helix). Unfortunately, there is no crystal structure for the human P-gp, a monomer. Based on the bacterial structure, the two (largely symmetric) segments constituting the NBD in the P-gp monomer can be swapping candidates. Such a swapping mechanism predicts that ribosome pausing occurring in the first transmembrane domain or in the first NBD would have no effect on the domain-swapping events; on the other hand, long-enough pauses following the translation of the first NBD segment and prior to the second can lead to nonswapped conformations. Thus, the native conformation may consist of either an ensemble of nonswapped and swapped species or only swapped species; the mutation-induced pausing will shift the landscape toward the nonswapped population. Such pauses can prevent domain-swapping events that are observed in the fast native folder. This may explain the difficulty in crystallizing the mixed population of the native conformation. Domain swapping effectively leads to kinetically trapped conformations.

## Conclusions: minima at the bottom of the funnel can have physiologically sufficiently high barriers leading to distinct conformations

Proteins are critical for all biological processes. To ensure proper function, they need to have the *correct* structure in the *right* population with a *favored* energy landscape. All the necessary data are en-

coded in the sequence; however, it is unclear how nature has engineered the protein folding mechanism and control. Recently, frequent-to-rare synonymous codon substitutions in the *MDR1* gene were implicated in at least two distinct P-gp conformations with altered functions. Rare codons lead to stalled ribosomes; hence, depending on the pause time scales, the upstream parts of the protein can fold prior to the downstream synthesis, affecting folding-pathway scenarios. P-gp is a transporter protein. Unfortunately, not much is known about the folding of membrane proteins as compared with globular proteins, and even less is known about their complex kinetics, which are likely to be affected by the interactions with the bilayer.[85]

The case of the *MDR1* gene provides a nice example of a mechanism that is likely to be used by nature to expand the functional repertoire. *MDR1* illustrates that stalling can lead to altered folding pathways, culminating in different minima in the folding funnel bottom. Yet, perhaps the most interesting point here is that while the structures are likely to possess only a limited conformational change and the free energy difference between them is likely to be small, the barriers are sufficiently high on physiological time scales, leading to the distinct conformational and functional states. Actually, in retrospect, this is not surprising: crystallization takes time due to the need to await identical conformers, in addition to the inherent improbability of bringing three, four, or five monomers together to form a viable nucleus. The surface is rough, and time is required to surmount the barriers. For the *MDR1* gene product, the fact that the conformers are distinct is observed from the different binding patterns of the Abs, the trypsin digestion, and the difference in the extent of inhibition by cyclosporine A and verapamil. Yet, it is also clear that the extent of the conformational change is not too large: native and polymorphic P-gp's are inhibited to similar extents by rapamycin.

Is nature using such a mechanism for function? We believe that this is the case for small conformational changes. P-gp is known to bind hundreds of substrates, and this mechanism may already operate there. While neither the structure of P-gp nor the locations of the SNP in the tertiary structure are unknown, it is rational to assume that even a small change in the membrane-buried part of the protein can lead to a large change in the extramembranous conformation, affecting the affinity of the mutant to the ligands. A conformational change in the membrane is expected to be small, since the membrane will not support a large change. There are numerous proteins used for multiple functions, particularly those with a range of ligands. The conformationally flexible *hub* proteins are likely to be a yet another example. Hubs are proteins with shared binding sites[86] that are critical in cellular networks, mediating numerous functions. They are frequently flexible, marginally stable, or disordered on their own. Yet, when crystallized in complex with partner proteins, they still assume a similar overall shape. Even in the binding site, the conformational difference can be remarkably small. On the other hand, a large conformational change under kinetic control is not a robust mechanism. Barrier heights are sensitive to environmental conditions, and over time, the proteins will flip into their thermodynamically most stable states. While there are proteins under kinetic control, like the proteases discussed above, such cases appear to be rare and designed for a specific function. The sensitivity of kinetic control to the environment is also indicated by the absence of dual-conformation cases in the Protein Data Bank and the lack of such observed cases of globally different conformations in folding–unfolding experiments. Protein sequences appear to be selected by evolution to avoid such traps.

We conclude that different folding pathways may lead to distinct minima at the bottom of the protein folding funnel, albeit with a small conformational change; nevertheless, the barrier heights, even if small, may be physiologically sufficiently high to lead to a new type of function or a conformational folding-pathway alteration. Similar mechanisms may also operate for proteins with shared binding sites; this would allow a smaller number of genes to perform an increasing number of functions. Distinct minima with small conformational changes may explain the more centralized nature of the cellular network and how central regulatory proteins are able to bind an astonishingly large number of different partners.

## Acknowledgements

The contents of this publication do not necessarily reflect the views or policies of the Department of Health and Human Services; neither does mention of trade names, commercial products, or organizations imply endorsement by the U.S. Government. This project has been funded in whole or in part with federal funds from the National Cancer Institute, National Institutes of Health, under contract number N01-CO-12400. This research was supported in part by the Intramural Research Program of the Center for Cancer Research of the National Cancer Institute, National Institutes of Health. We thank Dr. H. Robert Guy (Laboratory of Cell Biology, National Cancer Institute) for comments on the manuscript.

## References

1. Kimchi-Sarfaty, C., Oh, J. M., Kim, I. W., Sauna, Z. E., Calcagno, A. M., Ambudkar, S. V. & Gottesman, M. M. (2007). A "silent" polymorphism in the MDR1 gene changes substrate specificity. *Science*, **315**, 525–528.
2. Szakacs, G., Paterson, J. K., Ludwig, J. A., Booth-Genthe, C. & Gottesman, M. M. (2006). Targeting multidrug resistance in cancer. *Nat. Rev. Drug Discov.* **5**, 219–234.

3. Markova, S., Nakamura, T., Sakaeda, T., Makimoto, H., Uchiyama, H., Okamura, N. & Okumura, K. (2006). Genotype-dependent down-regulation of gene expression and function of MDR1 in human peripheral blood mononuclear cells under acute inflammation. *Drug Metab. Pharmacokinet.* **21**, 194–200.
4. Asano, T., Takahashi, K. A., Fujioka, M., Inoue, S., Okamoto, M., Sugioka, N. *et al.* (2003). ABCB1 C3435T and G2677T/A polymorphism decreased the risk for steroid-induced osteonecrosis of the femoral head after kidney transplantation. *Pharmacogenetics*, **13**, 675–682.
5. Tan, E. K., Chan, D. K. Y., Ng, P. W., Woo, J., Teo, Y. Y., Tang, K. *et al.* (2005). Effect of MDR1 haplotype on risk of Parkinson disease. *Arch. Neurol.* **62**, 460–464.
6. Pawlik, A., Baskiewicz-Masiuk, M., Machalinski, B., Kurzawski, M. & Gawronska-Szklarz, B. (2005). Involvement of C3435T and G2677T multidrug resistance gene polymorphisms in release of cytokines from peripheral blood mononuclear cells treated with methotrexate and dexamethasone. *Eur. J. Pharmacol.* **528**, 27–36.
7. Goreva, O. B., Grishanova, A. Y., Mukhin, O. V., Domnikova, N. P. & Lyakhovich, V. V. (2003). Possible prediction of the efficiency of chemotherapy in patients with lymphoproliferative diseases based on MDR1 gene G2677T and C3435T polymorphisms. *Bull. Exp. Biol. Med.* **136**, 183–185.
8. Sills, G. J., Mohanraj, R., Butler, E., McCrindle, S., Collier, L., Wilson, E. A. & Brodie, M. J. (2005). Lack of association between the C3435T polymorphism in the human multidrug resistance (MDR1) gene and response to antiepileptic drug treatment. *Epilepsia*, **46**, 643–647.
9. van den Heuvel-Eibrink, M. M., Wiemer, E. A. C., de Boevere, M. J., van der Holt, B., Vossebeld, P. J. M., Pieters, R. & Sonneveld, P. (2001). MDR1 gene-related clonal selection and P-glycoprotein function and expression in relapsed or refractory acute myeloid leukemia. *Blood*, **97**, 3605–3611.
10. Jamroziak, K., Mlynarski, W., Balcerczak, E., Mistygacz, M., Trelinska, J., Mirowski, M. *et al.* (2004). Functional C3435T polymorphism of MDR1 gene: an impact on genetic susceptibility and clinical outcome of childhood acute lymphoblastic leukemia. *Eur. J. Haematol.* **72**, 314–321.
11. Hesselink, D. A., van Schaik, R. H. N., van der Heiden, I. P., van der Werf, M., Gregoor, P., Lindemans, J. *et al.* (2003). Genetic polymorphisms of the CYP3A4, CYP3A5, and MDR-1 genes and pharmacokinetics of the calcineurin inhibitors cyclosporine and tacrolimus. *Clin. Pharmacol. Ther.* **74**, 245–254.
12. Zhu, D., Taguchi-Nakamura, H., Goto, M., Odawara, T., Nakamura, T., Yamada, H. *et al.* (2004). Influence of single-nucleotide polymorphisms in the multidrug resistance-1 gene on the cellular export of nelfinavir and its clinical implication for highly active antiretroviral therapy. *Antiviral Ther.* **9**, 929–935.
13. Kim, R. B., Fromm, M. F., Wandel, C., Leake, B., Wood, A. J. J., Roden, D. M. & Wilkinson, G. R. (1998). The drug transporter P-glycoprotein limits oral absorption and brain entry of HIV-1 protease inhibitors. *J. Clin. Invest.* **101**, 289–294.
14. Kafka, A., Sauer, G., Jaeger, C., Grundmann, R., Kreienberg, R., Zeillinger, R. & Deissler, H. (2003). Polymorphism C3435T of the MDR-1 gene predicts response to preoperative chemotherapy in locally advanced breast cancer. *Int. J. Oncol.* **22**, 1117–1121.
15. Ambudkar, S. V., Dey, S., Hrycyna, C. A., Ramachandra, M., Pastan, I. & Gottesman, M. M. (1999). Biochemical, cellular, and pharmacological aspects of the multidrug transporter. *Annu. Rev. Pharmacol. Toxicol.* **39**, 361–398.
16. Gribar, J. J., Ramachandra, M., Hrycyna, C. A., Dey, S. & Ambudkar, S. V. (2000). Functional characterization of glycosylation-deficient human P-glycoprotein using a vaccinia virus expression system. *J. Membr. Biol.* **173**, 203–214.
17. Loo, T. W. & Clarke, D. M. (1996). The minimum functional unit of human P-glycoprotein appears to be a monomer. *J. Biol. Chem.* **271**, 27488–27492.
18. Schumacher, M. A. & Brennan, R. G. (2003). Deciphering the molecular basis of multidrug recognition: crystal structures of the *Staphylococcus aureus* multidrug binding transcription regulator QacR. *Res. Microbiol.* **154**, 69–77.
19. Schumacher, M. A., Miller, M. C., Grkovic, S., Brown, M. H., Skurray, R. A. & Brennan, R. G. (2001). Structural mechanisms of QacR induction and multidrug recognition. *Science*, **294**, 2158–2163.
20. Schumacher, M. A., Miller, M. C., Grkovic, S., Brown, M. H., Skurray, R. A. & Brennan, R. G. (2002). Structural basis for cooperative DNA binding by two dimers of the multidrug-binding protein QacR. *EMBO J.* **21**, 1210–1218.
21. Yu, E. W., McDermott, G., Zgurskaya, H. I., Nikaido, H. & Koshland, D. E. (2003). Structural basis of multiple drug-binding capacity of the AcrB multidrug efflux pump. *Science*, **300**, 976–980.
22. Gottesman, M. M., Fojo, T. & Bates, S. E. (2002). Multidrug resistance in cancer: role of ATP-dependent transporters. *Nat. Rev. Cancer*, **2**, 48–58.
23. Tsai, C. J., Kumar, S., Ma, B. Y. & Nussinov, R. (1999). Folding funnels, binding funnels, and protein function. *Protein Sci.* **8**, 1181–1190.
24. Ma, B. Y., Kumar, S., Tsai, C. J. & Nussinov, R. (1999). Folding funnels and binding mechanisms. *Protein Eng.* **12**, 713–720.
25. Kumar, S., Ma, B. Y., Tsai, C. J., Sinha, N. & Nussinov, R. (2000). Folding and binding cascades: dynamic landscapes and population shifts. *Protein Sci.* **9**, 10–19.
26. Ma, B. Y. & Nussinov, R. (2002). Molecular dynamics simulations of alanine rich beta-sheet oligomers: insight into amyloid formation. *Protein Sci.* **11**, 2335–2350.
27. Rochet, J. C. & Lansbury, P. T. (2000). Amyloid fibrillogenesis: themes and variations. *Curr. Opin. Struct. Biol.* **10**, 60–68.
28. Fandrich, M. & Dobson, C. M. (2002). The behaviour of polyamino acids reveals an inverse side chain effect in amyloid structure formation. *EMBO J.* **21**, 5682–5690.
29. Clark, P. L. (2004). Protein folding in the cell: reshaping the folding funnel. *Trends Biochem. Sci.* **29**, 527–534.
30. Morrissey, M. P., Ahmed, Z. & Shakhnovich, E. I. (2004). The role of cotranslation in protein folding: a lattice model study. *Polymer*, **45**, 557–571.
31. Elcock, A. H. (2006). Molecular simulations of cotranslational protein folding: fragment stabilities, folding cooperativity, and trapping in the ribosome. *PLoS Comput. Biol.* **2**, 824–841.
32. Huard, F. P. E., Deane, C. M. & Wood, G. R. (2006). Modelling sequential protein folding under kinetic control. *Bioinformatics*, **22**, E203–E210.
33. Anfinsen, C. B. (1973). Principles that govern folding of protein chains. *Science*, **181**, 223–230.

34. Helenius, A. (1994). How N-linked oligosaccharides affect glycoprotein folding in the endoplasmic reticulum. *Mol. Biol. Cell*, **5**, 253–265.
35. Netzer, W. J. & Hartl, F. U. (1997). Recombination of protein domains facilitated by co-translational folding in eukaryotes. *Nature*, **388**, 343–349.
36. Tsai, C. J., Maizel, J. V. & Nussinov, R. (1999). Distinguishing between sequential and nonsequentially folded proteins: implications for folding and misfolding. *Protein Sci.* **8**, 1591–1604.
37. Wolynes, P. G., Onuchic, J. N. & Thirumalai, D. (1995). Navigating the folding routes. *Science*, **267**, 1619–1620.
38. Dill, K. A. & Chan, H. S. (1997). From Levinthal to pathways to funnels. *Nat. Struct. Biol.* **4**, 10–19.
39. Evans, M. S., Clark, T. F. & Clark, P. L. (2005). Conformations of co-translational folding intermediates. *Protein Pept. Lett.* **12**, 189–195.
40. Franke, A. E., Danley, D. E., Kaczmarek, F. S., Hawrylik, S. J., Gerard, R. D., Lee, S. E. & Geoghegan, K. F. (1990). Expression of human plasminogen activator inhibitor type-1 (PAI-1) in *Escherichia coli* as a soluble-protein comprised of active and latent forms. Isolation and crystallization of latent PAI-1. *Biochim. Biophys. Acta*, **1037**, 16–23.
41. Reiss, C., Lesnik, T., Parvez, H., Parvez, S. & Ehrlich, R. (2000). Conformational toxicity and sporadic conformational diseases. *Toxicology*, **153**, 115–121.
42. Purvis, I. J., Bettany, A. J. E., Santiago, T. C., Coggins, J. R., Duncan, K., Eason, R. & Brown, A. J. P. (1987). The efficiency of folding of some proteins is increased by controlled rates of translation *in vivo*—a hypothesis. *J. Mol. Biol.* **193**, 413–417.
43. Crombie, T., Boyle, J. P., Coggins, J. R. & Brown, A. J. P. (1994). The folding of the bifunctional Trp3 protein in yeast is influenced by a translational pause which lies in a region of structural divergence with *Escherichia coli* indoleglycerol-phosphate synthase. *Eur. J. Biochem.* **226**, 657–664.
44. Komar, A. A., Lesnik, T. & Reiss, C. (1999). Synonymous codon substitutions affect ribosome traffic and protein folding during *in vitro* translation. *FEBS Lett.* **462**, 387–391.
45. Cortazzo, P., Cervenansky, C., Marin, M., Reiss, C., Ehrlich, R. & Deana, A. (2002). Silent mutations affect *in vivo* protein folding in *Escherichia coli*. *Biochem. Biophys. Res. Commun.* **293**, 537–541.
46. Dong, H. J., Nilsson, L. & Kurland, C. G. (1996). Co-variation of tRNA abundance and codon usage in *Escherichia coli* at different growth rates. *J. Mol. Biol.* **260**, 649–663.
47. Ivanov, I. G., Saraffova, A. A. & Abouhaidar, M. G. (1997). Unusual effect of clusters of rare arginine (AGG) codons on the expression of human interferon alpha 1 gene in *Escherichia coli*. *Int. J. Biochem. Cell Biol.* **29**, 659–666.
48. Chamary, J. V., Parmley, J. L. & Hurst, L. D. (2006). Hearing silence: non-neutral evolution at synonymous sites in mammals. *Nat. Rev. Genet.* **7**, 98–108.
49. Akashi, H. (2003). Translational selection and yeast proteome evolution. *Genetics*, **164**, 1291–1303.
50. Dyer, R. B. (2007). Ultrafast and downhill protein folding. *Curr. Opin. Struct. Biol.* **17**, 38–47.
51. Plaxco, K. W., Simons, K. T. & Baker, D. (1998). Contact order, transition state placement and the refolding rates of single domain proteins. *J. Mol. Biol.* **277**, 985–994.
52. Ma, B. Y., Tsai, C. J. & Nussinov, R. (2000). Binding and folding: in search of intramolecular chaperone-like building block fragments. *Protein Eng.* **13**, 617–627.
53. Thanaraj, T. A. & Argos, P. (1996). Protein secondary structural types are differentially coded on messenger RNA. *Protein Sci.* **5**, 1973–1983.
54. Thanaraj, T. A. & Argos, P. (1996). Ribosome-mediated translational pause and protein domain organization. *Protein Sci.* **5**, 1594–1612.
55. Duret, L. & Mouchiroud, D. (1999). Expression pattern and, surprisingly, gene length shape codon usage in *Caenorhabditis*, *Drosophila*, and *Arabidopsis*. *Proc. Natl Acad. Sci. USA*, **96**, 4482–4487.
56. Sorensen, M. A. & Pedersen, S. (1991). Absolute *in vivo* translation rates of individual codons in *Escherichia coli*—the 2 glutamic acid codons GAA and GAG are translated with a threefold difference in rate. *J. Mol. Biol.* **222**, 265–280.
57. Curran, J. F. & Yarus, M. (1989). Rates of aminoacyl-trans-RNA selection at 29 sense codons *in vivo*. *J. Mol. Biol.* **209**, 65–77.
58. Sorensen, M. A., Kurland, C. G. & Pedersen, S. (1989). Codon usage determines translation rate in *Escherichia coli*. *J. Mol. Biol.* **207**, 365–377.
59. Fox, J. D., Routzahn, K. M., Bucher, M. H. & Waugh, D. S. (2003). Maltodextrin-binding proteins from diverse bacteria and archaea are potent solubility enhancers. *FEBS Lett.* **537**, 53–57.
60. Baker, D., Silen, J. L. & Agard, D. A. (1992). Protease pro-region required for folding is a potent inhibitor of the mature enzyme. *Proteins: Struct., Funct., Genet.* **12**, 339–344.
61. Shinde, U. & Inouye, M. (1994). The structural and functional organization of intramolecular chaperones—the N-terminal propeptides which mediate protein folding. *J. Biochem. (Tokyo)*, **115**, 629–636.
62. Shinde, U. P., Liu, J. J. & Inouye, M. (1997). Protein memory through altered folding mediated by intramolecular chaperones. *Nature*, **389**, 520–522.
63. Tsai, C. J., Ma, B. Y. & Nussinov, R. (1999). Folding and binding cascades: shifts in energy landscapes. *Proc. Natl Acad. Sci. USA*, **96**, 9970–9972.
64. Batey, S. & Clarke, J. (2006). Apparent cooperativity in the folding of multidomain proteins depends on the relative rates of folding of the constituent domains. *Proc. Natl Acad. Sci. USA*, **103**, 18113–18118.
65. Klausner, R. D., Kempf, C., Weinstein, J. N., Blumenthal, R. & Vanrenswoude, J. (1983). The folding of ovalbumin—renaturation *in vitro versus* biosynthesis *in vitro*. *Biochem. J.* **212**, 801–810.
66. Komar, A. A., Kommer, A., Krasheninnikov, I. A. & Spirin, A. S. (1997). Cotranslational folding of globin. *J. Biol. Chem.* **272**, 10646–10651.
67. Crombie, T., Swaffield, J. C. & Brown, A. J. P. (1992). Protein folding within the cell is influenced by controlled rates of polypeptide elongation. *J. Mol. Biol.* **228**, 7–12.
68. Kleizen, B., van Vlijmen, T., de Jonge, H. R. & Braakman, I. (2005). Folding of CFTR is predominantly cotranslational. *Mol. Cell*, **20**, 277–287.
69. Du, K., Sharma, M. & Lukacs, G. L. (2005). The F508 cystic fibrosis mutation impairs domain–domain interactions and arrests post-translational folding of CFTR. *Nat. Struct. Mol. Biol.* **12**, 17–25.
70. Lukacs, G. L., Mohamed, A., Kartner, N., Chang, X. B., Riordan, J. R. & Grinstein, S. (1994). Conformational maturation of CFTR but not its mutant counterpart (Delta-F508) occurs in the endoplasmic reticulum and requires ATP. *EMBO J.* **13**, 6076–6086.
71. Ward, C. L. & Kopito, R. R. (1994). Intracellular turnover of cystic fibrosis transmembrane conductance regulator—inefficient processing and rapid

degradation of wild-type and mutant proteins. *J. Biol. Chem.* **269**, 25710–25718.
72. Varga, K., Jurkuvenaite, A., Wakefield, J., Hong, J. S., Guimbellot, J. S., Venglarik, C. J. *et al.* (2004). Efficient intracellular processing of the endogenous cystic fibrosis transmembrane conductance regulator in epithelial cell lines. *J. Biol. Chem.* **279**, 22578–22584.
73. Schumacher, M. A., Miller, M. C. & Brennan, R. G. (2004). Structural mechanism of the simultaneous binding of two drugs to a multidrug-binding protein. *EMBO J.* **23**, 2923–2930.
74. Murray, D. S., Schumacher, M. A. & Brennan, R. G. (2004). Crystal structures of QacR–diamidine complexes reveal additional multidrug-binding modes and a novel mechanism of drug charge neutralization. *J. Biol. Chem.* **279**, 14365–14371.
75. Nakatsukasa, K. & Brodsky, J. L. (2008). The recognition and retrotranslocation of misfolded proteins from the endoplasmic reticulum. *Traffic*, **9**, 861–870.
76. Tsai, B., Ye, Y. H. & Rapoport, T. A. (2002). Retro-translocation of proteins from the endoplasmic reticulum into the cytosol. *Nat. Rev. Mol. Cell Biol.* **3**, 246–255.
77. Buck, T. M., Wright, C. M. & Brodsky, J. L. (2007). The activities and function of molecular chaperones in the endoplasmic reticulum. *Semin. Cell Dev. Biol.* **18**, 751–761.
78. Tsai, C. J., Maizel, J. V. & Nussinov, R. (2000). Anatomy of protein structures: visualizing how a one-dimensional protein chain folds into a three-dimensional shape. *Proc. Natl Acad. Sci. USA*, **97**, 12038–12043.
79. Tsai, C. J. & Nussinov, R. (2001). The building block folding model and the kinetics of protein folding. *Protein Eng.* **14**, 723–733.
80. Bennett, M. J., Schlunegger, M. P. & Eisenberg, D. (1995). 3D domain swapping—a mechanism for oligomer assembly. *Protein Sci.* **4**, 2455–2468.
81. Bennett, M. J., Choe, S. & Eisenberg, D. (1994). Domain swapping—entangling alliances between proteins. *Proc. Natl Acad. Sci. USA*, **91**, 3127–3131.
82. Dalessio, G. (1995). Oligomer evolution in action. *Nat. Struct. Biol.* **2**, 11–13.
83. Piccoli, R., Tamburrini, M., Piccialli, G., Didonato, A., Parente, A. & Dalessio, G. (1992). The dual-mode quaternary structure of seminal RNase. *Proc. Natl Acad. Sci. USA*, **89**, 1870–1874.
84. Dawson, R. J. P. & Locher, K. P. (2006). Structure of a bacterial multidrug ABC transporter. *Nature*, **443**, 180–185.
85. White, S. H. & von Heijne, G. (2005). Do protein–lipid interactions determine the recognition of transmembrane helices at the ER translocon? *Biochem. Soc. Trans.* **33**, 1012–1015.
86. Keskin, O. & Nussinov, R. (2007). Similar binding sites and different partners: implications to shared proteins in cellular pathways. *Structure*, **15**, 341–354.

*Note added in proof*: After acceptance of our manuscript, we become aware of a related paper in press (Evans, AM, Sander, IM, Clark PL: Contranslational folding promotes beta-helix formation and avoids aggregation in vivo, JMB (2008) doi:10.1016/j.jmb.2008.07.035). The authors observed that conformations accessible to P22 tailspike nascent chains tethered to the ribosome (and even after ribosome release) are distinct from the conformations adopted by free polypeptide chains denatured in urea and refolded by delution. This distinction is independent of the presence or absence of the ribosome-bound chaperone trigger factor. These observations provide direct experimental support for our proposal that co-translational folding can lead to distinct energy minima.
