# Supplementary material for: Computational Analysis of MDR1 Variants Predicts Effect on Cancer Cells via their Effect on mRNA Folding
Source: PLoS Comput Biol. 2024 Dec 26;20(12):e1012685. doi: 10.1371/journal.pcbi.1012685 (PMC11670953; doi:10.1371/journal.pcbi.1012685)
Supplement: Attachment — Submitted filename: Response to reviewersTT.pdf [file pcbi.1012685.s020.pdf]

Dear Prof. Mohammad Sadegh Taghizadeh,

Please find enclosed the revised version of our manuscript now entitled 'Computational Analysis of MDR1 Variants Predicts Effect on Cancer Cells via their Effect on mRNA Folding'. We are happy that the reviews were very positive about our work, and recommended its publication, after adjustments required. In the revised version, we answered issues raised by the reviewers; see details hereafter.

We hope you find the current reversion of the paper satisfactory.

Below please find our detailed answers (in blue) to the comments we received (in black). The changes in the manuscript are highlighted.

Sincerely yours,

Prof. Tamir Tuller

On behalf of all authors

Response to reviewers:

Editors' notes:

Dear Dr. Tuller,

Thank you very much for submitting your manuscript "Evidence that MDR1 Variants affect cancer cells via their effect on mRNA folding" for consideration at PLOS Computational Biology. As with all papers reviewed by the journal, your manuscript was reviewed by members of the editorial board and by several independent reviewers. The reviewers apreciated the attention to an important topic. Based on the reviews, we are likely to accept this manuscript for publication, providing that you modify the manuscript according to the review recommendations.

Please prepare and submit your revised manuscript within 30 days. If you anticipate any delay, please let us know the expected resubmission date by replying to this email.

When you are ready to resubmit, please upload the following:

[1] A letter containing a detailed list of your responses to all review comments, and a description of the changes you have made in the manuscript. Please note while forming your response, if your article is accepted, you may have the opportunity to make the peer review history publicly available. The record will include editor decision letters (with reviews) and your responses to reviewer comments. If eligible, we will contact you to opt in or out

[2] Two versions of the revised manuscript: one with either highlights or tracked changes denoting where the text has been changed; the other a clean version (uploaded as the manuscript file).

Important additional instructions are given below your reviewer comments.

Thank you again for your submission to our journal. We hope that our editorial process has been constructive so far, and we welcome your feedback at any time. Please don't hesitate to contact us if you have any questions or comments.

Sincerely,

Mohammad Sadegh Taghizadeh, Ph.D.

Academic Editor

PLOS Computational Biology

Ilya Ioshikhes

Section Editor

PLOS Computational Biology

Thank you. Below please find our detailed answers (in blue) to the comments we received (in black). A version of the manuscript with changes highlighted was also submitted.

Reviewer #1:

This paper uses computational approaches to re-explore the effect of three specific single nucleotide polymorphisms which occur commonly in the ABCB1 (MDR1 or

ABCB1) gene. There have been many studies relating these single polymorphisms and combinations of them to clinical responses to chemotherapy (with conflicting results), and several papers using in vitro models (not all referenced here) showing that the synonymous polymorphism T3435C affects substrate specificity of the ABCB1 transporter through mechanisms related to protein and/or mRNA folding. Although this polymorphism is predicted to change the folding of mRNA, neither the previous papers nor this one provides direct evidence of such an effect. Thus, the title of the paper is misleading. Given that this is an area that has achieved a great deal of attention over many years, does this paper add additional information to the existing literature?

1. The data in Fig. 1 based on clinically available data indicate that the three polymorphisms under study are not associated with statistically significant changes in expression of ABCB1. This is one of the most definitive demonstrations in the literature of this observation.
2. Since polymorphisms in theory could affect mRNA splicing, and many examples have been found in other proteins of effects on splicing of SNPs, the lack of association with splicing variants is useful and novel information.
3. The analysis of codon usage is interesting and applicable to other systems.
4. The data showing a potential association of T1236C with patient outcomes is more problematic since it isn't combined with detailed information about the tumors, their chemotherapy (?substrates transported by ABCB1), or toxicity information (a major function of ABCB1 is handling of drugs in the GI tract, kidney, and liver). In fairness to the authors, they indicate that this association needs further analysis including tumor and drug stratification.
5. The prediction that T3435C increases the local rate of translation suggests that translation rate could affect kinetics of protein folding at bottlenecks and, in theory, might explain effects on ABCB1 protein function. This is interesting, though in the absence of experimental verification, is hypothetical.
6. The authors apply their analysis to many other diseases resulting from genetic alterations and make several predictions about association of specific polymorphisms with severity of disease and/or presentation. Absent specific experimental verification, it is difficult to know whether these predictions are accurate and whether they represent a self-fulfilling hypothesis or can actually predict outcomes.
7. In the Discussion the authors refer to what appears to be a very relevant paper by Gottesman and Leung Fung as reference [76] but there are only 20 references in this paper and this paper does not appear in the reference list. Additional relevant references which should be discussed are attached.

Given the several new contributions this paper makes to the literature, especially the novel way of predicting "translatability" this contribution does appear to be significant. However, there should be more reference and Discussion of the existing literature which addresses the same issue (see above, point #7). In addition, the authors confuse computational prediction with experimental verification, including in the title "Evidence that MDR1 Variants affect cancer cells via their effect on mRNA folding." There is no

such “evidence” in the paper. The title should be “Computational Prediction that MDR1 Variants affect cancer cells via their effect on mRNA folding.”

Thank you for your positive review of our contribution.

Thank you for providing these valuable additional references. We have revised the discussion section to incorporate a review of these references and now provide a more detailed analysis of existing work in this field. As for the inconsistencies in the bibliography, we are not sure what the problem was as we see 82 references in the submitted version and proof. We now make sure that all the references appear in the proof.

We appreciate the important point about the distinction between computational predictions and experimental evidence. We have revised the title to more accurately reflect the nature of our work: "Computational Analysis of MDR1 Variants Predicts Effect on Cancer Cells via their effect on mRNA Folding". Furthermore, we have carefully reviewed the entire manuscript to ensure our language appropriately reflects the computational nature of our findings. This includes:

1. Adding appropriate caveats where needed (for example, in the description of Figure 11)
2. Including a clear statement in the Discussion section about the limitations of computational predictions and the need for experimental validation

This change better represents the scope and methodology of our study while maintaining scientific rigor.

Have the authors made all data and (if applicable) computational code underlying the findings in their manuscript fully available?
Reviewer #1: **No:** Cannot evaluate. They refer to a technique not previously published and not described in enough detail to allow replication.

We have now added a very extensive description of the co-translational folding model to Supporting Information File 1, under Supporting Methods. To make it clearer, we incorporate examples and also illustrate major steps of the model in the new figures S8 and S9. Additionally, we also provide the code in a website dedicated to the paper: https://www.cs.tau.ac.il/~tamirtul/Co-Trans-FE/

Reviewer #2:

The manuscript “Evidence that MDR1 Variants affect cancer cells via their effect on mRNA folding” by Tal Gutman and Tamir Tuller is an *in silico* analysis of the potential biological effects of the MDR1 variants T1236C, T2677G, and T3435C. The study is

very extensive, covering most potential mRNA and translational effects caused by the MDR1 variants.

Thank you for the positive feedback.

I have listed minor points that can be additive to the analysis presented in this manuscript:

Page 13 (statisticalsignificance p = 0.08) seems to be the continuation of the incomplete sentence at page 12 that was incorrectly spaced.

Fixed, thank you.

Are there any studies in the literature that validate the *in silico* conclusion of the MDR1 variants in it's mRNA expression and protein abundance?

The literature regarding the effects of these three MDR1 variants on mRNA expression and protein abundance is mixed, with several studies supporting our in-silico predictions and others contradicting them. Supporting our findings, Kimchi-Sarfaty et al.[1] and K. L. Fung et al.[2] reported no significant changes in both mRNA and protein levels in their respective cell models. Additionally, J. M. Gow et al.[3] and N. N. Salama et al.[4] found no effects on either mRNA expression or protein abundance, respectively. However, other studies[5], [6], [7], [8], [9] suggest that at least some of these variants do impact mRNA expression or protein abundance under various conditions and in different tissues or cell lines. Given these conflicting experimental results, we conducted our in-silico analysis to provide additional insights and contribute to this ongoing debate in the field.

We have now addressed this important point and cited these papers in the third paragraph of the introduction and in the eighth paragraph of the discussion.

For the progression free survival curves, it is not clear if the authors took all cancer types from the TCGA to construct the curve. MDR1 progression free survival may differ for each cancer type. Have the authors performed the progression free survival on the cancers in which the MDR1 variants are more frequent? This analysis would add more context and reason to explore MDR1 variants in those cancers.

Several issues arise from this question-

1. We recognize that our original presentation was confusing - the curves that were previously shown were overall survival curves and not progression-free survival curves. However, we acknowledge the importance of PFS curves as well and have now added them to the revised manuscript (see Figures 6, S3, S5 and S6).

2. In the main figure shown in the Results section (Figure 6), all TCGA cancer types were used in the analysis. However, in Figures S5-S6 in supplemental file 1 we stratify patients according to groups of cancer types. We added a clarification about this in the methods section, under “Survivability of TCGA patients” (2.7).
3. As for conducting the survival analyses for specific cancer types, and specifically those for which the MDR1 variants are more common, this is challenging with the current dataset due to the small number of mutated carriers. For example, this is the distribution of cancer types for carriers of the somatic mutation T1236C in TCGA –

Cancer Type	Number of Carriers of T1236C
LUAD	4
SARC	3
ACC	2
GBM	2
KIRP	2
DLBC	2
STAD	1
OV	1
BLCA	1
THCA	1
UCEC	1

The same situation occurs for T2677G and T3435C. As can be seen, there are very few patients in the mutated cohort for each cancer type. Given these small sample sizes, conducting statistically robust analyses for individual cancer types is unfortunately not feasible at this time. We fully agree that cancer type significantly influences survival outcomes, and we hypothesize that analyzing specific types could reveal even stronger associations. Our approach of grouping related cancer types (Figures S5-S6) was an attempt to balance these considerations while maintaining statistical power. We now discuss this topic in the seventh paragraph of the discussion section.

The authors mentioned in the discussion of the potential of post-translational modifications mediating the effects of MDR1 variants. Have the authors considered the possibility of post-transcriptional modifications changes in MDR1 mRNA due to the folding changes caused by the variants? As an example, A-to-I editing occurs at double stranded RNA structures (see the review Cheng H. et. al. *Protein & cell*, 2024). Given the changes in mRNA structure, the MDR1 variants might change A-to-I editing sites.

While our initial analysis of post-transcriptional modifications focused solely on splicing, we agree that the variants' effect on the mRNA secondary structure could impact additional post-transcriptional mechanisms. Therefore, we have now expanded our investigation to include potential effects on A-to-I editing and m6A modifications, two prevalent post-transcriptional modifications in eukaryotes that can be influenced by mRNA structural changes.

To assess potential changes in these modifications, we employed two predictive models:

1. Airliner[10] for A-to-I editing sites, analyzing sequences of 1001 nucleotides centered on each variant position
2. DeepM6ASeq[11] for m6A modifications, using sequences of 101 nucleotides (the maximum length supported by the model) centered on each variant position

For each variant, we compared the reference and mutated sequences, defining significant changes as positions where the modification probability shifted by more than 50% in either direction.

While these models don't explicitly incorporate mRNA secondary structure as a feature, they can implicitly capture structural influences through their analysis of the extended sequence context. We attempted to identify prediction tools that directly incorporate secondary structure information but found none currently available for use.

Our analyses revealed no significant alterations in either A-to-I editing sites or m6A modification patterns due to the MDR1 variants. In fact, none of the site probabilities changes by more than 5% and in most cases much less than that.

We have incorporated these new analyses into the revised manuscript, including the methodology (section 2.3) and results (section 3.2).

Additionally, have the authors explored whether the mRNA structural changes of the variants disrupt any motifs recognized by RNA binding proteins in the vicinity of the mutation? RNA binding proteins at the CDS can alter transcriptional stability and rate of translation.

We addressed this question by investigating potential disruptions to RNA binding protein (RBP) interactions using the catRAPID omics model[12]. This model is particularly suitable as it explicitly incorporates mRNA secondary structure, along with other molecular features including hydrogen bonding and Van der Waals interactions, in its prediction of mRNA-protein binding probabilities.

For each variant, we analyzed both reference and mutated sequences to identify potential RBP binding sites. We defined significant changes as cases where the z-score difference between reference and variant sequences exceeded one standard deviation, indicating a substantial change in binding probability. Our analysis revealed no significant alterations in RBP binding patterns for any of the variants examined.

These findings have been integrated into the manuscript under the same sections as our other post-transcriptional modification analyses (methodology in section 2.3 and results in section 3.2).

Reviewer #3:

I will try to be brief The paper is excellent The authors studied the MDR1 T1236C, T2677G, and T3435C polimorfisms and the variants for the significantly changes the mRNA folding in their vicinity. This change in mRNA structure is predicted to increase local translation elongation rates, but not to change the protein expression Importantly the increased translation rate near T3435C is predicted to affect the protein's co-translational folding trajectory in the region of the second ATP binding domain. This potentially impacts protein conformation and function.
The importance of this paper besides of being very solid is that the authors have used a computational approach to show molecular mechanisms of MDR1 variants and their potential impact on cancer prognosis and treatment resistance. But this is the tip of the iceberg on how this approach could be used for other proteins Last they have demonstrated the importance to identify mutations affecting mRNA folding in pathology.

Thank you for the very positive feedback.

I have the suggestion that the authors could improve this paper to explain why they have only showed the Kaplan Meier only for overall survival but not for the failure-free survival...

We did not conduct a progression-free survival analysis because we did not have clinical data related to the progression and recurrence of the TCGA patients' disease. However, following this suggestion, we examined other resources and encountered clinical data on cBioPortal[13] (https://www.cbioportal.org/) that contained more extensive clinical information for the same TCGA patients in our analysis. This data also contained the information needed to construct the PFS curves, and so we have now added this analysis to the manuscript. See revised images Figure 6 in the main text and Figures S3, S5 and S6 in supplemental file 1.

References

[1] C. Kimchi-Sarfaty *et al.*, "A 'Silent' Polymorphism in the MDR1 Gene Changes Substrate Specificity," *Science*, vol. 315, pp. 525–528, Feb. 2007, doi: 10.1126/science.1135308.

[2] K. L. Fung and M. M. Gottesman, "A synonymous polymorphism in a common MDR1 (ABCB1) haplotype shapes protein function," *Biochimica et Biophysica Acta (BBA) - Proteins and Proteomics*, vol. 1794, no. 5, pp. 860–871, 2009, doi: https://doi.org/10.1016/j.bbapap.2009.02.014.

[3] J. M. Gow, L. M. Hodges, L. W. Chinn, and D. L. Kroetz, "Substrate-dependent effects of human ABCB1 coding polymorphisms.," *J Pharmacol Exp Ther*, vol. 325, no. 2, pp. 435–442, May 2008, doi: 10.1124/jpet.107.135194.

[4] N. N. Salama, Z. Yang, T. Bui, and R. J. Y. Ho, "MDR1 haplotypes significantly minimize intracellular uptake and transcellular P-gp substrate transport in recombinant LLC-PK1 cells.," *J Pharm Sci*, vol. 95, no. 10, pp. 2293–2308, Oct. 2006, doi: 10.1002/jps.20717.

[5] H. He *et al.*, "Association of ABCB1 polymorphisms with prognostic outcomes of anthracycline and cytarabine in Chinese patients with acute myeloid leukemia.," *Eur J Clin Pharmacol*, vol. 71, no. 3, pp. 293–302, Mar. 2015, doi: 10.1007/s00228-014-1795-6.

[6] S. J. Hemauer, T. N. Nanovskaya, S. Z. Abdel-Rahman, S. L. Patrikeeva, G. D. V Hankins, and M. S. Ahmed, "Modulation of human placental P-glycoprotein expression and activity by MDR1 gene polymorphisms.," *Biochem Pharmacol*, vol. 79, no. 6, pp. 921–925, Mar. 2010, doi: 10.1016/j.bcp.2009.10.026.

[7] S. Hoffmeyer *et al.*, "Functional polymorphisms of the human multidrug-resistance gene: multiple sequence variations and correlation of one allele with P-glycoprotein expression and activity in vivo.," *Proc Natl Acad Sci U S A*, vol. 97, no. 7, pp. 3473–3478, Mar. 2000, doi: 10.1073/pnas.97.7.3473.

[8] P. Song *et al.*, "G2677T and C3435T genotype and haplotype are associated with hepatic ABCB1 (MDR1) expression," *J Clin Pharmacol*, vol. 46, pp. 373–379, 2006, doi: 10.1177/0091270005284387.

[9] L. Pang *et al.*, "ATP-Binding Cassette Genes Genotype and Expression: A Potential Association with Pancreatic Cancer Development and Chemoresistance?," *Gastroenterol Res Pract*, vol. 2014, p. 414931, 2014, doi: 10.1155/2014/414931.

[10] G. Nigita, S. Alaimo, A. Ferro, R. Giugno, and A. Pulvirenti, “Knowledge in the Investigation of A-to-I RNA Editing Signals,” *Front Bioeng Biotechnol*, vol. 3, Mar. 2015, doi: 10.3389/fbioe.2015.00018.
[11] Y. Zhang and M. Hamada, “DeepM6ASeq: prediction and characterization of m6A-containing sequences using deep learning,” *BMC Bioinformatics*, vol. 19, no. 19, p. 524, 2018, doi: 10.1186/s12859-018-2516-4.
[12] F. Agostini, A. Zanzoni, P. Klus, D. Marchese, D. Cirillo, and G. Tartaglia, “CatRAPID omics: A web server for large-scale prediction of protein-RNA interactions,” *Bioinformatics*, vol. 29, Nov. 2013, doi: 10.1093/bioinformatics/btt495.
[13] E. Cerami *et al.*, “The cBio Cancer Genomics Portal: An Open Platform for Exploring Multidimensional Cancer Genomics Data,” *Cancer Discov*, vol. 2, pp. 401–404, May 2012, doi: 10.1158/2159-8290.CD-12-0095.
