# Supplementary material for: Computational Analysis of MDR1 Variants Predicts Effect on Cancer Cells via their Effect on mRNA Folding
Source: PLoS Comput Biol. 2024 Dec 26;20(12):e1012685. doi: 10.1371/journal.pcbi.1012685 (PMC11670953; doi:10.1371/journal.pcbi.1012685)
Supplement: Attachment — Submitted filename: reviewer comments.docx [file pcbi.1012685.s019.docx]

The manuscript “Evidence that MDR1 Variants affect cancer cells via their effect on mRNA folding” by Tal Gutman and Tamir Tuller is an *in silico* analysis of the potential biological effects of the MDR1 variants T1236C, T2677G, and T3435C. The study is very extensive, covering most potential mRNA and translational effects caused by the MDR1 variants. I have listed minor points that can be additive to the analysis presented in this manuscript:

1- Page 13 (statisticalsignificance p = 0.08) seems to be the continuation of the incomplete sentence at page 12 that was incorrectly spaced.
2- Are there any studies in the literature that validate the *in silico* conclusion of the MDR1 variants in it’s mRNA expression and protein abundance?
3- For the progression free survival curves, it is not clear if the authors took all cancer types from the TCGA to construct the curve. MDR1 progression free survival may differ for each cancer type. Have the authors performed the progression free survival on the cancers in which the MDR1 variants are more frequent? This analysis would add more context and reason to explore MDR1 variants in those cancers.
4- The authors mentioned in the discussion of the potential of post-translational modifications mediating the effects of MDR1 variants. Have the authors considered the possibility of post-transcriptional modifications changes in MDR1 mRNA due to the folding changes caused by the variants? As an example, A-to-I editing occurs at double stranded RNA structures (see the review Cheng H. et. al. *Protein & cell*, 2024). Given the changes in mRNA structure, the MDR1 variants might change A-to-I editing sites.
5- Additionally, have the authors explored whether the mRNA structural changes of the variants disrupt any motifs recognized by RNA binding proteins in the vicinity of the mutation? RNA binding proteins at the CDS can alter transcriptional stability and rate of translation.
